# Deconstruction of Vermal Cerebellum in Ramp Locomotion in Mice

**DOI:** 10.1002/advs.202203665

**Published:** 2022-11-14

**Authors:** Chenfei Lyu, Chencen Yu, Guanglong Sun, Yue Zhao, Ruolan Cai, Hao Sun, Xintai Wang, Guoqiang Jia, Lingzhu Fan, Xi Chen, Lin Zhou, Ying Shen, Lixia Gao, Xinjian Li

**Affiliations:** ^1^ Department of Neurology of the Second Affiliated Hospital and Interdisciplinary Institute of Neuroscience and Technology Zhejiang University School of Medicine Hangzhou 310027 China; ^2^ Department of Physiology and Department of Psychiatry Sir Run Run Shaw Hospital Zhejiang University School of Medicine Hangzhou 310058 China; ^3^ Key Laboratory for Biomedical Engineering of Ministry of Education College of Biomedical Engineering and Instrument Science, Zhejiang University Hangzhou 310027 China; ^4^ Department of Neuroscience City University of Hong Kong Kowloon Hong Kong China; ^5^ MOE Frontier Science Center for Brain Science and Brain‐machine Integration School of Brain Science and Brain Medicine Zhejiang University Hangzhou 310027 China; ^6^ Key Laboratory of Medical Neurobiology of Zhejiang Province Hangzhou 310027 China

**Keywords:** cerebellum, downhill  locomotion, molecular interneurons, Purkinje cells, ramp walking, uphill  locomotion

## Abstract

The cerebellum is involved in encoding balance, posture, speed, and gravity during locomotion. However, most studies are carried out on flat surfaces, and little is known about cerebellar activity during free ambulation on slopes. Here, it has been imaged the neuronal activity of cerebellar molecular interneurons (MLIs) and Purkinje cells (PCs) using a miniaturized microscope while a mouse is walking on a slope. It has been found that the neuronal activity of vermal MLIs specifically enhanced during uphill and downhill locomotion. In addition, a subset of MLIs is activated during entire uphill or downhill positions on the slope and is modulated by the slope inclines. In contrast, PCs showed counter‐balanced neuronal activity to MLIs, which reduced activity at the ramp peak. So, PCs may represent the ramp environment at the population level. In addition, chemogenetic inactivation of lobule V of the vermis impaired uphill locomotion. These results revealed a novel micro‐circuit in the vermal cerebellum that regulates ambulatory behavior in 3D terrains.

## Graphical abstract

By imaging the neuronal activity of cerebellar molecular interneurons and Purkinje cells, it has been found that the neuronal activity of MLIs specifically enhances during uphill and downhill locomotion and PCs show counter‐balanced neuronal activity to MLIs. These results reveal a novel micro‐circuit in the vermal cerebellum that regulates ambulatory behavior in 3D terrains.

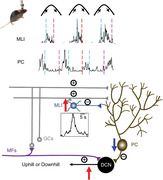

## 1 Introduction

The cerebellum is widely recognized to receive and integrate external and internal sensorimotor contextual signals during movement.^[^ ^1^, ^2^, ^3^, ^4^, ^5^ ^]^ By integrating afferent signals from the spinal cord^[^ ^6^ ^]^ and vestibular system,^[^ ^7^ ^]^ the cerebellum participates in controlling head movement,^[^ ^8^ ^]^ balance,^[^ ^9^ ^]^ posture,^[^ ^10^ ^]^ and gravity^[^ ^11^, ^12^, ^13^ ^]^ during locomotion. Anatomically, the cerebellar cortex has distinctive laminar structures and neuronal organization,^[^ ^14^ ^]^ including the granular layer, the Purkinje layer, and the molecular layer.^[^ ^14^ ^]^ Molecular layer interneurons (MLIs) receive afferent signals from granule cells (GCs) and inhibit Purkinje cells (PCs) to modulate motor behaviors.^[^ ^15^ ^]^ In vivo two‐photon imaging in head‐fixed mice has demonstrated that MLIs encode the licking rate through coherent neuronal activity^[^ ^16^ ^]^ and valence during associative learning.^[^ ^17^ ^]^ PCs display time‐locked activity to skilled movements^[^ ^18^ ^]^ and determine the timing of sensory‐evoked motor initiation.^[^ ^19^ ^]^ GCs display heterogeneous activity and acquire predictive signals during forelimb^[^ ^20^ ^]^ or eyelid movement.^[^ ^5^ ^]^ In addition, MLIs exhibit enhanced neuronal activity during locomotion than during still conditions,^[^ ^15^, ^21^, ^22^ ^]^ and PCs involve in the step‐cycle of locomotion.^[^ ^23^, ^24^ ^]^ However, due to technical limitations, the encoding of ambulation in the micro‐circuit of the superficial layer of the cerebellum in freely moving conditions, especially on a ramp, remains largely unknown.

Although our daily movement usually takes place in 3D environments, most movement‐related studies have been carried out on flat surfaces, especially those on the striatum,^[^ ^25^, ^26^ ^]^ cortex,^[^ ^20^, ^27^, ^28^ ^]^ and cerebellum.^[^ ^8^, ^20^ ^]^ How the brain perceives and coordinates locomotion on a ramp remains poorly understood. As one of the most common locomotor behaviors in daily life, walking up and down has been studied in terms of energy consumption^[^ ^29^, ^30^ ^]^ and postural adaptation,^[^ ^31^ ^]^ but the underlying neural mechanisms remain unclear. In the current study, we established an ambulation paradigm of the mouse walking up and down on a slope and adopted a strategy to successfully visualize the neuronal activity in the superficial layer of the cerebellum with a miniature microscope, which allowed us to investigate how the cerebellum is involved in locomotion on a ramp. Strikingly, we found vermal MLIs are activated during uphill and downhill walking, and the animal's locomotion during ramp walking could be decoded by MLIs activity in the vermis. However, PCs displayed counter‐balanced neural activity to the MLIs and may represent the ramp environment at the population level. Taken together, our results demonstrate that a micro‐circuit in the superficial layer of the cerebellum is crucial for ramp walking, which regulates ambulatory behaviors in 3D terrain.

## 2 Results

### 2.1 Imaging MLIs Activity in Freely‐Behaving Mice

**Figure 1:**
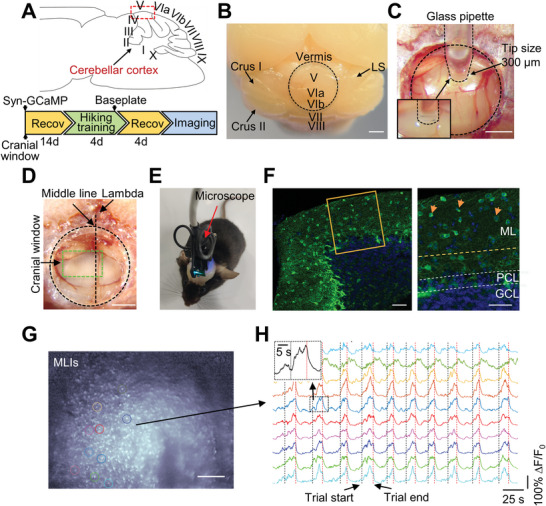
Imaging MLIs activity in the superficial layer of the vermis in freely‐behaving mice. A) Upper: sketch showing the viral labeling regions and imaged lobules of the cerebellum (red box). Lower: outline of the experimental procedure. Recov: recovery after surgery from virus injection or baseplate placement. B) Dorsal view of the cerebellum. Lobule V in the cerebellar vermis was infused with AAV‐GCaMP6 (black dotted circle, brain for chronic window; scale bar, 1 mm). C) Example image showing cerebellar surface‐based viral infusion using a glass pipette with a tip size of 0.3–0.5 mm (scale bar, 1 mm). D) Example image showing clear blood vessels inside the cranial window 21 days after virus infusion and window implantation (green dotted rectangle, imaged area; scale bar, 1 mm). E) Representative Ca^2+^ imaging with a miniature microscope in a freely‐behaving mouse. F) Left: Confocal fluorescent image of a coronal cerebellar section showing labeled interneuron and a few Purkinje neurons in the superficial layer using the surface‐based viral (AAV2/9‐hSyn‐GCaMP6s, green) infusion method. Right: the enlarged image of the yellow box in the left panel. Interneurons are indicated by orange arrows (scale bars, 50 µm). ML, molecular layer; PCL, Purkinje cell layer; GCL, granule cell layer. Neuron nuclei were labeled by DAPI (blue). G) Single‐frame ∆*F*/*F*_0_ image showing neurons captured by a miniature microscope (scale bar, 100 µm). The ∆*F*/*F*_0_ image is included through the standard deviation projection of 20 000 ∆*F*/*F*_0_ frames. H) Example traces demonstrating the Ca^2+^ transients of individual neurons from ROIs with corresponding colors of neurons in (G). Black and red dotted lines denote the start and end of individual trials, respectively. The subpanel on the upper left is the enlarged plot of the region in the rectangle.

To assess neuronal activity in the molecular layer of the cerebellum, we adopted an imaging strategy that was previously established to visualize neuronal activity in the cortical superficial layer.^[^ ^32^ ^]^ First, we infused GCaMP6 into the cerebellum using a glass pipette (tip size: 0.3 mm, **Figure** **1**A–C), which allowed us to label the most superficial interneurons in the cranial window (lobule V in the vermis; Figure 1A,B,F, and Figure S1A, Supporting Information). Second, we placed a cranial window on the top of the vermis (Figure 1D), which allowed us to image the activity of a large number of cerebellar neurons using a miniature microscope at single‐cell resolution in freely‐moving mice without overt damage to the cerebellum (Figure 1E,G,H, and Video S1, Supporting Information). The focal plane of the microscope was set to 70 µm from the pial surface, which was in the molecular interneuron layer (Figure 1F). We also tried to image GCs with a cell‐specific marker (math1‐cre mice infected with flexed‐GCaMP6) using a miniscope, but no neurons with single‐cell resolution were captured (data not shown). Purkinje cells could be distinguished from MLIs by a bigger soma or striped dendrites during imaging.^[^ ^19^, ^33^, ^34^ ^]^ Therefore, based on somal size, target shapes and imaged depth, most imaged neurons were classified as MLIs and few neurons with bigger somata and striped dendrites were excluded from analysis (see Section 4). Furthermore, the imaged neurons exhibited concerted neural activity during the locomotor task (Figure 1H, Video S1, Supporting Information), similar to the activity pattern of MLIs imaged through a two‐photon microscope.^[^ ^16^, ^35^ ^]^

### 2.2 MLIs Activity Elevated During Uphill and Downhill Locomotion

**Figure 2:**
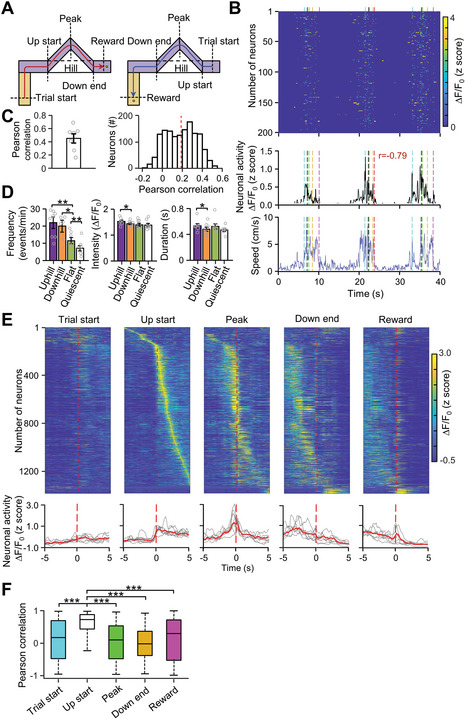
MLIs activity was elevated during uphill and downhill locomotion. A) Diagram showing the ramp‐walking events used for neuronal Ca2+ activity alignment in right trials (red) and left trials (blue). The behavioral events in each trial are indicated by vertical dashed lines. The trial start is defined as the time when a mouse receives a reward in the last trial, turns around and tries to start a new trial; Upstart is defined as the time when the mouse arrives at the ramp and gets ready to climb up; Peak refers to the moment when the mouse (center of the body) reaches the top of the ramp; Down end refers to the moment when the mouse arrives the end of the ramp and its hindlimb just leave the ramp; Reward refers to the time when the mouse reaches the food well. B) Upper: Ca^2+^ event activity of imaged cells during the uphill and downhill locomotion task of an example mice in the L maze. Each row indicates the Ca^2+^ events in a single neuron. Middle: Averaged Ca^2+^ activity of all recorded neurons (lower) over time (n =216) from an example mouse during uphill and downhill locomotion. Lower, the speed of the example animal. Dotted lines indicate movement locations and phases [cyan, trial movement start; black, uphill start; green, peak; yellow, downhill end; red (left trials) and pink (right trials), reward]. *r*, Pearson correlation between normalized MLIs activity and locomotion speed of mice. C) Pearson correlation between MLIs activity and speed of mice at the population level (left) and the distribution of Pearson correlation for at individual neuron level (right). D) Averaged Ca^2+^ event frequency (left, one‐way ANOVA, *F* = 5.81, *p* < 0.01), event intensity (middle, one‐way ANOVA, *F* = 1.14, *p* = 0.35) and event duration (right, one‐way ANOVA, *F* = 0.86, *p* = 0.48) in uphill, downhill, flat walking, and quiescence from seven mice. Quiescence is defined by the time between the reward end and the next trial initiation. *p < 0.05; **p < 0.01 (one‐way ANOVA followed by paired Student's *t*‐test). E) Upper: trial‐averaged neuronal activity of the MLIs during ramp locomotion. The Ca^2+^ event activity in different trials is aligned with different movement locations or behaviors (dashed red vertical line): trial start (1st row), uphill beginning (2nd row), top of the ramp (3rd row), downhill end (4th row), and reward start (5th row). All task‐related traces are averaged from all trials (24 ± 3 trials per mouse) and sorted based on their peak activation time during the uphill beginning and displayed in temporal raster plots (1487 neurons in 7 mice). Lower: averaged Ca^2+^ responses of MLIs aligned by the five behaviors events (red curves). Light gray curves indicate the Ca^2+^ responses of the individual animal. F) Pearson correlation between MLIs activity and mice speed at different phases of locomotion. The MLIs activity and transient speed were calculated from 1 s before and 1 s after the behavior events. (one‐way ANOVA, *F* = 224, *p* < 0.001).

To examine MLIs activity on a ramp, we designed a behavioral paradigm, an L‐shaped maze (L maze) that included a long flat arm, a short flat arm, and two ramps (up and down, **Figure** **2**A and Figure S1B, Supporting Information) to mimic climbing behavior during hiking and going upstairs. In this paradigm, mice were required to pass through a level and two ramps to receive a food pellet reward placed at either end (Figure 2A). Accordingly, mice went many round trips and their behaviors were divided into four phases (uphill, downhill, flat, and quiescent) based on their location inside the L maze (Figure S1B, Supporting Information). The results showed that MLIs' activity was strongly correlated with mice locomotion behaviors both at the population level and single neuron level (Figure 2B,C). Interestingly, the activity was generally higher during ramp walking than that during level walking and quiescent periods (Figure 2D). To further identify the encoding patterns of the cerebellum in different phases of walking, the neuronal activity of individual imaged neurons was aligned with individual movement events: trial movement start, uphill start, reaching the ramp peak, downhill end, and reward (Figure 2A,E, Figures S1C, S2, and S3, Supporting Information). Our analysis showed that neuronal activity in the vermis exhibited phase specificity: some neurons started to fire at the beginning of uphill; some neurons showed maximal activity at the peak; and some stopped firing at the end of downhill (Figure 2E, Figures S1–S3, Supporting Information). In addition, the MLIs' activity at uphill start was strongly correlated with the animal's locomotion velocity (Figure 2F). Furthermore, to cross‐validate our results, we compared the MLIs activity of the first half trials with that of the second half trials. We found that MLIs showed similar activity patterns in the first half trials to those in the second half trials in all event conditions (Figure S3, Supporting Information). In addition, a small proportion of imaged neurons showed higher activity when animals received a food pellet (Figure 2E, Figures S2 and S3, Supporting Information), consistent with reward‐related responses in the vermis from previous studies.^[^ ^36^, ^37^, ^38^, ^39^, ^40^ ^]^

### 2.3 Phase‐Specific Neuronal Activity of the Vermal MLIs During Ramp Walking

**Figure 3:**
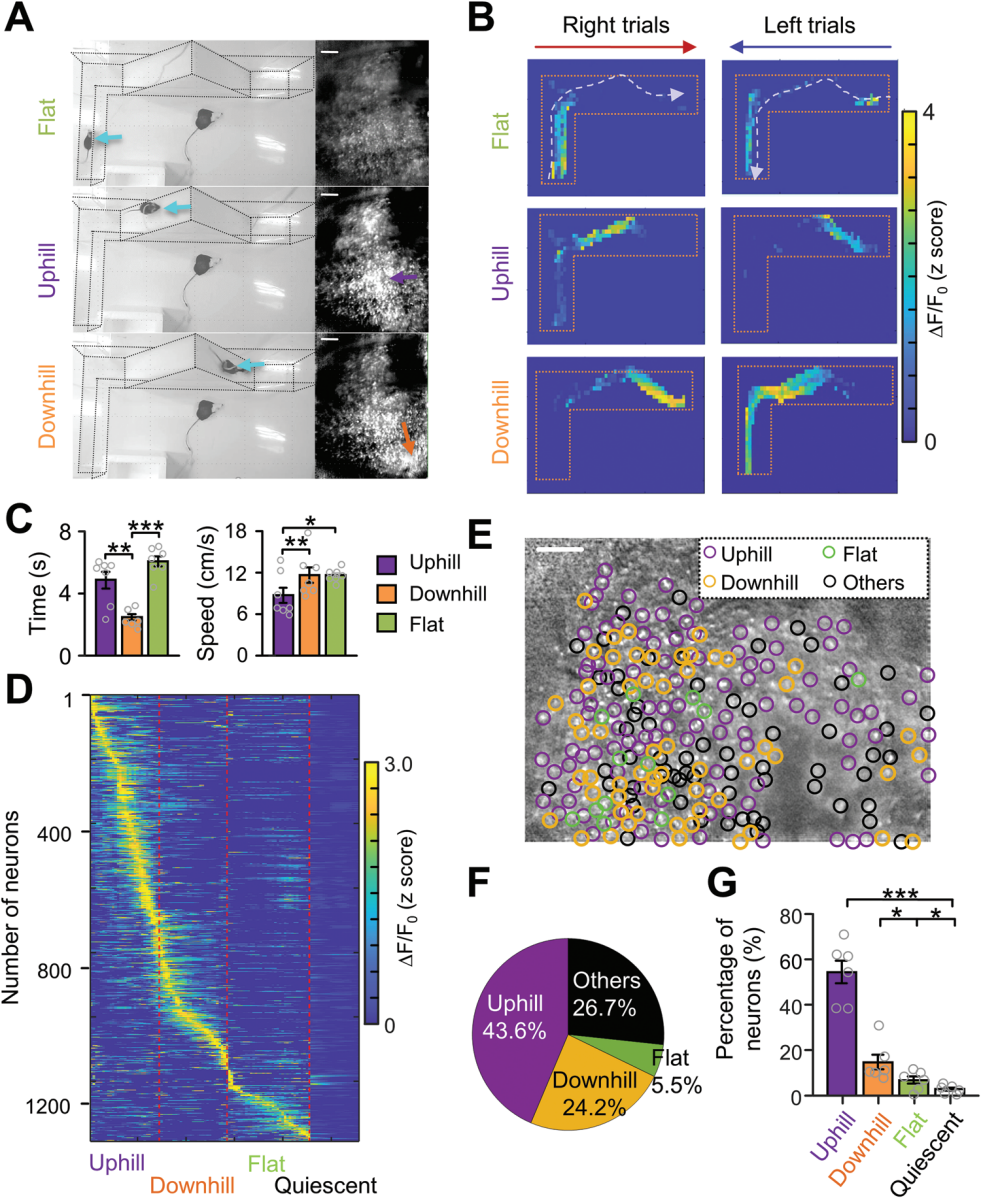
Phase‐specific neuronal activity of vermis in ramp walking. A) Left: video frames showing the location of a mouse in the L maze (cyan arrows) while the mouse on flat walking (upper), uphill (middle), and downhill (lower). Right: Sampled Ca^2+^ movie frames showing neuronal activity corresponding to movement phases on the left. Ca^2+^ signals are expressed as ∆*F*/*F*_0_ (*F*_0_ is the averaged Ca^2+^ activity of >15 000 frames). Scale bars, 100 µm. B) Heatmap demonstrating neuronal activity patterns of three types of neurons firing at three movement phases in an L maze. Upper: flat walking neuron; middle: uphill neuron; lower: downhill neuron. The Ca^2+^ activity of the three sampled neurons is correlated with movement states regardless of left or right trials. C) Averaged time (left, one‐way ANOVA, *F* = 18.96, *p* < 0.001) and speed (right, one‐way ANOVA, *F* = 2.2, *p* = 0.14) in uphill, downhill, and flat walking from seven mice. *p < 0.05; **p < 0.01, ***p < 0.001 (one‐way ANOVA followed by paired Student's *t*‐test). D) Trial‐averaged Ca^2+^ activity during uphill downhill, flat walking, and quiescence period of 1487 MLIs in seven mice. The trial‐averaged activity was sorted based on their peak activation time during four different stages. Most neurons displayed higher activity during ramp walking. Quiescence was defined as 1s after reward during which the animals were kept still on the flat surface. E) Distribution of uphill, downhill, and flat walking neurons in an example imaging area of the cerebellum. All the detected neurons (*n* = 236) in the imaging field are included through the standard deviation projection of 20 000 ∆*F*/*F*_0_ frames, and the activation preference of individual neurons is labeled by a colored circle according to movement phase (purple: uphill; orange: downhill; green: flat walking; and black: others). Others defined the MLIs activity during reward and turning. Scale bar, 100 µm. F) Pie chart showing the percentage of uphill, downhill, and flat walking neurons from the example mouse in (D). G) Percentages of uphill, downhill, flat walking, and quiescent neurons in all recorded animals (7 animals and 1478 neurons) *p < 0.01; ***p < 0.001 (one‐way ANOVA followed by paired Student's test, *F* = 20.98, *p* < 0.001).

To investigate whether the vermis has a spatial organization in different locomotion contexts, the Ca^2+^ activity of individual neurons was analyzed during three movement stages: uphill, downhill, and flat (**Figure** **3**A,B). We found that individual neurons exhibited phase‐specific Ca^2+^ activity during flat (Figure 3B, top), uphill (Figure 3B, middle), or downhill (Figure 3B, bottom) stages, no matter whether mice started trials from the left end or the right end of the maze. These results indicated that neuronal activity was strongly correlated with movement stages but irrelevant to the initial position and horizontal direction in the L maze (Figure 3B). Next, we analyzed the running time and speed during uphill, downhill, and flat walking phases and found that the mice displayed longer walking time and slower velocity during uphill than downhill walking (Figure 3C). To further clarify the ramp‐related neurons, we compared the MLI's activity during uphill, downhill, flat, and quiescent periods (1 s after reward, flat surface still) and found most neurons displayed higher activity during ramp walking (Figure 3D). In our study, an uphill neuron was defined by the criterion when the activity of the neuron during uphill walking was not only significantly higher than that during quiescent and downhill walking periods but also was the highest among all locations in the L maze. The downhill and flat walking neurons were defined by similar methods and showed the highest activity in the downhill location or flat walking region, respectively. Spatially, uphill neurons were broadly dispersed in the imaged area and downhill neurons intermingled with the uphill neurons (Figure 3E). Out of 1487 imaged neurons recorded from seven mice, 63.1% of neurons (Figure 3F,G; uphill, 46.9% and downhill, 16.2%) increased activity during uphill or downhill locomotion. We further selected the trials with similar running time and velocity during uphill and downhill walking phases, and we found that there are more uphill neurons than downhill neurons in these trials as well (Figure S4, Supporting Information). These results indicated that the uphill was over‐represented in the vermal cerebellum during the ramp locomotion.

**Figure 4:**
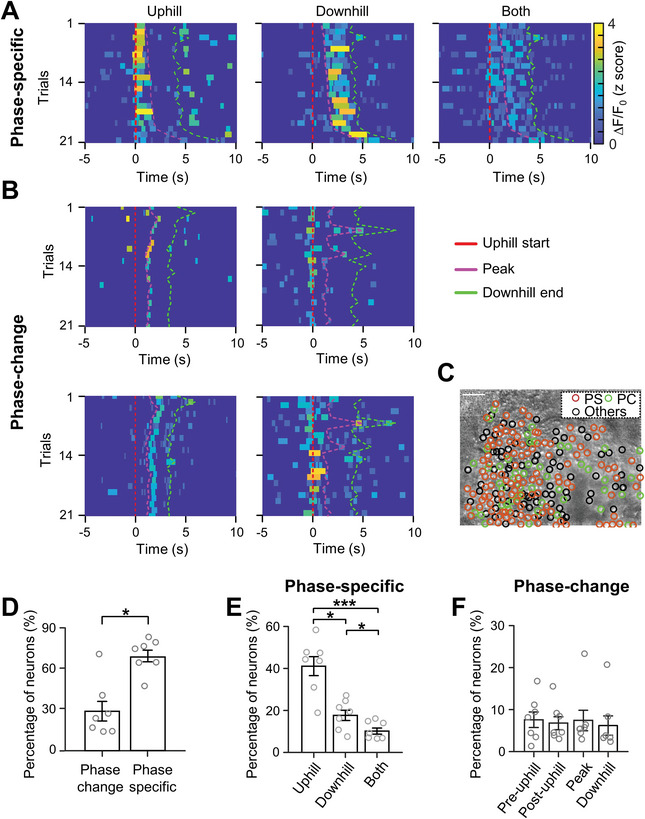
Heterogeneous MLI activity during uphill or downhill walking on a ramp. A) Heatmaps demonstrating Ca^2+^ activity of 3 example phase‐specific neurons: uphill (left), downhill (middle), and both (right) across different trials. The activity is aligned by “up start” (red line) and sorted by the relative time that the mouse reached the ramp “peak” (pink line). The down end is labeled by dashed green lines. The uphill neuron is activated over the entire uphill walking and the downhill neuron is activated over the entire downhill position regardless of left or right trials. The activity of “Both”‐type neurons covers both uphill and downhill locations. B) Heatmap showing 4 example phase‐change neurons. The neurons show higher activity during the transition from uphill to downhill (top left); from level to uphill (top right); both from uphill to downhill and downhill to level (bottom, left); both from level to uphill and downhill to level (bottom, right). C) Distribution of phase‐specific and phase‐change neurons in an example imaging area of the cerebellum. All the detected neurons (*n* = 236) in the imaging field are included through the standard deviation projection of 20 000 ∆*F*/*F*_0_ frames, and the activation preference of individual neurons is labeled by a colored circle according to activity pattern (brown: phase‐specific neurons; green: phase‐change neurons, and black: others) (scale bar, 100 µm). D) Percentages of phase‐specific and phase‐change neurons in all recorded animals (7 animals and 1478 neurons; *p < 0.05, Student's *t*‐test). E) Percentages of uphill, downhill, and both types of phase‐specific neurons. (one‐way ANOVA followed by student *t*‐test, *F* = 23.39, *p* < 0.001) ***, *p* < 0.001; *, *p* < 0.05. F) Percentages of phase‐change neurons which activated before uphill, after uphill, peak, and downhill of the slope.

To further dissect whether the cerebellum responds to the whole movement phases or phase changes (for example: from level to uphill or uphill to downhill, Figure S1C, Supporting Information left and middle), we aligned the Ca^2+^ activity of individual neurons with “uphill start” in each trial (**Figure** **4**) and found that the Ca^2+^ activity of some neurons selectively spanned over “uphill,” “downhill,” or both phases (Figure 4A), although the running duration and horizontal direction differed between even and odd trials. In addition, some neurons showed transient activity at the position during single (Figure 4B, upper) or combined movement phase changes (Figure 4B, lower). Statistically, 68% phase‐specific and 27% phase‐change neurons were dispersed in lobule V of the vermis (Figure 4C,D). Furthermore, the majority of phase‐specific neurons were identified as uphill neurons (Figure 4E) and a similar proportion of phase‐change neurons were found at different transition points of uphill, slope peak, and downhill (Figure 4F). These results indicate that the vermis encodes ramp locomotion in both phase‐specific and phase‐change manner.

### 2.4 MLIs Activity is Elevated during Uphill and Downhill Locomotion Regardless of Movement Direction and Ramp Location

**Figure 5:**
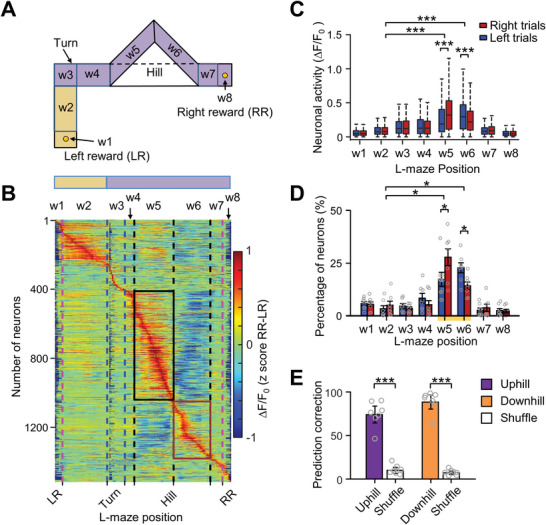
MLIs activity was elevated during uphill and downhill locomotion regardless of movement direction and ramp location. A) Sketch showing the eight segments of the L maze representing different motion behaviors (see Section 4) in the task. The L maze includes one horizontal arm (purple) and one vertical arm (yellow). B) Pseudo‐color map showing the subtraction of neuronal Ca^2+^ activity at each location in the L maze between right and left trials for all recorded neurons. The activity difference between right and left trials is projected into 130 bins in the L maze and then sorted based on their peak activation location. Notably, in the right trials, w5 represents uphill walking and w6 represents downhill walking; it is opposite in the left trials. Clearly, a larger group of neurons in the superficial vermis showed positive activity in w5 (black rectangle) and negative activity in w6 which indicates the uphill neurons. Another group was downhill neurons (brown rectangle). C) Average Ca^2+^ activity in eight segments of the L maze for all imaged neurons of seven mice in left and right trials (*n* = 1478 neurons, two‐way ANOVA followed by Bonferroni's test for multiple comparisons, *F* = 45.98, *p* < 0.001). ***p < 0.001. D) Proportions of neurons in the vermis of seven mice exhibiting task‐related activation in eight segments of the L maze. There is a higher proportion of uphill neurons than downhill neurons (*F* = 14.29, *p* < 0.001). *p < 0.05. E) Prediction correction of uphill and downhill locomotion using the support vector machine (SVM) algorithm (two‐way ANOVA followed by Bonferroni's test for multiple comparisons, *F* = 124.48, *p* < 0.001), ***p < 0.001.

To further verify that the MLIs respond to the locomotion or the location in the arena, we projected the MLI's activity of left or right trials into the L maze (**Figure** **5**A and Figure S5, Supporting Information). In this analysis, “w5” represented uphill locations in right trials and downhill locations in left trials, while “w6” represented downhill locations in right trials and uphill locations in left trials. Our results showed that the MLIs exhibited the highest Ca^2+^ activity during uphill walking irrespective of movement direction or location (Figure S5, Supporting Information). Moreover, different MLIs were activated at different movement phases in a sequence that may modulate PC activity promptly (Figure S5, Supporting Information). We also subtracted the neuronal activity in right trials from those in left trials in the same neural populations (Figure 5B). A large number of neurons showed higher positive activity in “w5” and negative activity in “w6” (Figure 5B), demonstrating higher neuronal activity during uphill walking in both right and left trials. A similar trend was found in neurons encoding downhill walking (Figure 5B). These results further support the idea that the MLIs represent either uphill or downhill walking, irrespective of the location in the arena. Statistical analyses showed higher activity (Figure 5C) as well as a larger proportion (Figure 5D) of MLIs during ramp‐related movements.

To further confirm the importance of MLIs during ramp walking, we applied a support vector machine (SVM) algorithm^[^ ^41^, ^42^ ^]^ to classify the neuronal signals and predict movement phases related to ramp locomotion. First, we selected half trials randomly to train an SVM classifier, in which animals' behavior at specific time points was grouped into three phases: uphill, downhill, and others. Then, we applied the trained SVM classifier to the neuronal activity of the remaining trials, and the results revealed that the accuracy of the SVM classifier was much higher during both uphill and downhill on the ramp than that for the shuffled data (Figure 5E). These results further confirmed that the MLIs in vermis encoded uphill and downhill walking.

### 2.5 Vermal MLIs Encode Slope Incline during Ramp Locomotion

**Figure 6:**
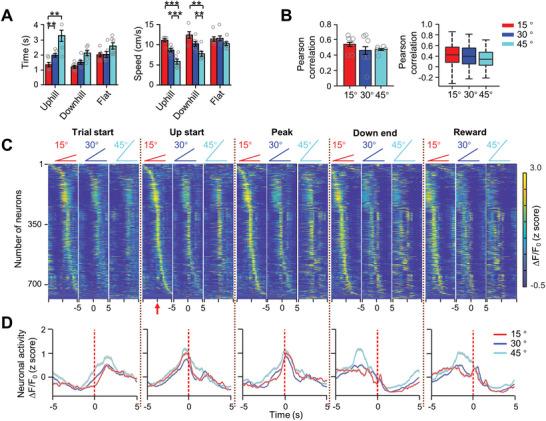
Vermal MLIs encode slope inclines during ramp locomotion. A) Averaged time (left, one‐way ANOVA, *F* = 9.98, *p* < 0.001) and speed (right, two‐way ANOVA, *F* = 15.1, *p* < 0.001) in uphill, downhill and flat walking from six mice on three slopes (15°, 30°, and 45°). *p < 0.05; **p < 0.01, ***p < 0.001 (one‐way ANOVA followed by paired Student's *t*‐ test). B) Pearson correlation between MLIs activity and speed at different slope inclines at the population level (left, one‐way ANOVA, *F* = 0.49, *p* = 0.62) and individual neuron level (right). C) Comparison of MLIs (807 MLIs from 6 mice) activity under three slope inclines conditions (15°, 30°, and 45°). Averaged responses of MLIs were aligned to five behavioral events and sorted based on peak activation time during the beginning of uphill on the 15° slope. 15° slope: height, 6.42 cm, length, 48 cm; 30° slope: height, 13.9 cm, length, 48 cm; 45° slope: height, 24 cm, length, 48 cm. Red arrow: event and slope for neural sorting sequence. D) Averaged neuronal responses of 807 MLIs aligned to five different behavior events. Red, 15° slope; blue, 30° slope; Cyan, 45° slope.

**Figure 7:**
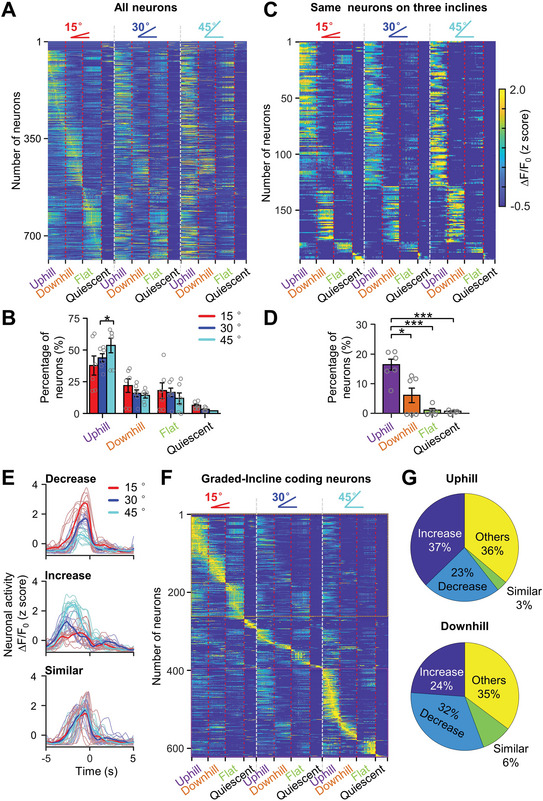
Vermal MLIs encode slope inclines in different manners. A) The comparison of trial‐averaged Ca^2+^ activity at uphill, downhill, flat walking, and quiescence phases from 807 MLIs of six mice when they ran on 15°, 30°, and 45° slopes, respectively. The activity was sorted based on peak activation time on the 15° slope. Left, 15° slope; middle, 30° slope; Right; 45° slope. B) Proportion of uphill, downhill, flat walking, and quiescent neurons when the mice ran on 15°, 30°, and 45° slopes. Two‐way ANOVA followed by Bonferroni's test for multiple comparisons, *F* = 11.81, *p* < 0.001. *p < 0.05; Red, 15° slope; blue, 30° slope; cyan, 45° slope. C) Pseudo‐color map showing stable uphill, downhill, flat walking, and quiescence neurons at all three15°, 30°, and 45° slopes (*n* = 190 neurons from 6 mice). D) Proportion of MLIs with stable activation at uphill, downhill, flat walking, and quiescence phases on 15°, 30°, and 45° slopes (one‐way ANOVA followed by paired Student's *t*‐test, *F* = 17.944, *p* < 0.001). *p < 0.05; ***p < 0.001. E) Ten sampled neurons with decreased activity when slope inclines increased (Upper), with increased activity when slope inclines increased (Middle), and with similar responses at three different inclines (Bottom). Trial‐averaged neuronal activities were aligned to the time when mice reached the peak of the ramp. Thicker lines represent the averaged neuronal activity of ten sampled neurons. Thinner lines represent the trial‐averaged activity of individual neurons. Red, MLIs activity on 15° slope; Blue, MLI activity on 30° slope; Cyan, MLI activity on 45° slope. F) Pseudo‐color map showing incline‐encoding neurons (*n* = 617 neurons from 6 mice). Upper light‐yellow box, MLIs activity increased with increasing inclines; Lower magenta box, MLIs activity decreased with increasing inclines. G) Pie chart showing the percentages of MLIs (*n* = 617 neurons from 6 mice) with increased, decreased, and similar activity on 15°, 30°, and 45° slope inclines. Upper, uphill phase; Bottom, downhill phase. Others indicate the neurons in response to one slope or no response to any slope.

To further verify whether MLIs encode slope incline, we imaged MLI's activity at 15°, 30°, and 45° slopes with another batch of mice. We found that the mice decreased velocity and increased the walking time when the slope inclines increased (**Figure** **6**A). In addition, a similar Pearson correlation coefficient was found between MLIs activity and movement speed at different inclines both at the population level and at the individual neuron level (Figure 6B). Next, we aligned MLI's activity with the time of uphill moving start and sorted by the maximal activation at the 15° slope (Figure 6C, red arrow). The MLIs sequences in other behavior events and other slope inclines were the same as those in 15° slope at uphill start. We found that the MLIs showed higher activation during slope walking than during flat walking and quiescent period no matter they were walking on 15°, 30°, and 45° slope inclines (Figure 6C). Generally, vermal MLIs showed a similar degree of activation at different inclines when activity was aligned to the same behavioral events (Figure 6C). We also compared MLIs activity during uphill, downhill, flat walking and quiescence among three slope inclines (15°, 30°, and 45°) at the population level and found that MLIs displayed similar activation patterns under three slope inclines (Figure 6D). Interestingly, we found the proportion of uphill neurons gradually increased with increasing inclines (**Figure** **7**A,B). In addition, we found that some neurons with higher activity during flat walking than other phases in a small slope incline displayed higher activity during uphiill walking at the 45° slope condition. This result indicated that some neurons representing flat‐walking in smaller slope conditions changed to slope‐activated neurons in the higher slope condition (Figure 7A). Statistically, 22.5% of neurons consistently exhibited maximal activity on uphill or downhill slopes under three incline conditions (Figure 7C,D). Furthermore, the neural activity of uphill‐ and downhill‐activated neurons was not correlated with animals' running direction (Figure S6, Supporting Information). These results indicated that the vermal MLIs were stably discharged during ramp locomotion, regardless of the size of the inclines.

To further elucidate whether MLIs can differentiate the slope inclines, we compared individual MLIs' activity under the three inclines (Figure 7E,F). We found that some neurons displayed increased neuronal activity with increasing slope inclines, some neurons displayed decreased neuronal activity with increasing slope inclines (Figure 7E,F), and some neurons showed similar activity among three slope inclines (Figure 7C,E). Interestingly, more MLIs increased their activities with increasing slope inclines at the uphill phase. In contrast, more MLIs decreased neuronal activity with increasing slope inclines at the downhill phase (Figure 7G). These results suggest that vermal MLIs encode slope inclines in different manners.

### 2.6 PCs Exhibited Counter‐Balance Neuronal Activity to MLIs During Ramp Locomotion

**Figure 8:**
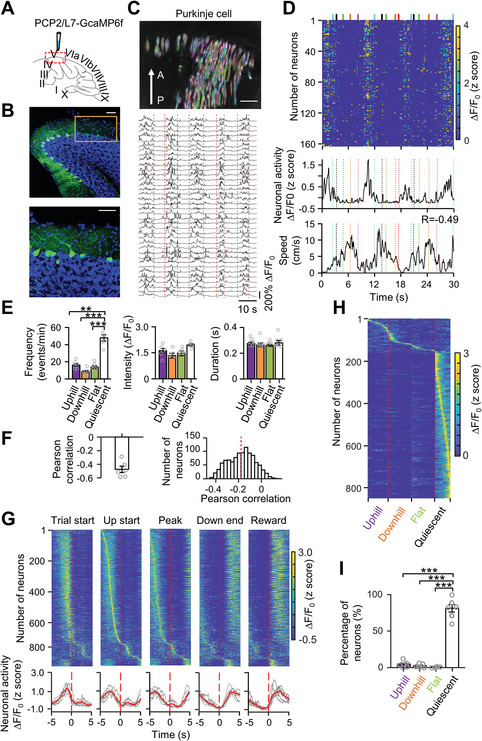
Vermal PCs displayed induced activity during ramp locomotion. A) Sketch showing the viral labeling location of PCs by AAV2/8‐PCP2/L7‐GCaMP6f. B) Upper: confocal fluorescence image showing PCs specifically labeled by AAV2/8‐PCP2/L7‐GCaMP6f. Lower: enlargement of PCs in the Upper. Scale bar, 50 µm. C) Upper: Single‐frame ∆*F*/*F*_0_ image showing PCs captured by a miniature microscope (scale bar, 100 µm). The ∆*F*/*F*_0_ image was included through the standard deviation projection of 20 000 ∆*F*/*F*_0_ frames. Colored stripe region: ROIs of PCs. Lower: Example traces of Ca^2+^ transients of individual PCs from ROIs in upper. D) Upper: Ca^2+^ events of PCs during uphill and downhill locomotion tasks in L‐maze. Each row indicates Ca^2+^ events in a single PC. Middle: Average Ca^2+^ activity of all recorded neurons (upper) over time from example mouse during uphill and downhill walking. Lower: average speed (lower) of the example mice. Dotted lines indicate movement locations and phases [cyan, trial movement start; black, uphill start; green, peak; yellow, downhill end; red (left trials); and pink (right trials), reward]. E) Average Ca^2+^ event frequency (left), event intensity (middle), and event duration (right) during uphill, downhill, flat walking, and quiescence from six mice. Quiescence was defined as the time between the reward end and the next trial initiation. The animal kept still on the flat surface. **p < 0.01; ***p < 0.001 (one‐way ANOVA followed by paired Student's *t‐*test). F) Pearson correlation between PCs activity and speed at the population level (left) and individual neuron level (right). G) Upper: trial‐averaged neuronal activity of PCs (839 PCs from 6 mice) in lobule V during ramp locomotion. Ca^2+^ event activity in different trials was aligned to different movement locations or behaviors (dashed red vertical line): trial start (1st row), uphill beginning (2nd row), the peak of the ramp (3rd row), downhill end (4th row), and reward start (5th row). All task‐related traces were averaged from all trials (58 ± 7 trials), sorted based on peak activation time during the uphill beginning, and displayed in temporal raster plots (839 PCs in 6 mice). Lower: Average Ca^2+^ responses of 839 PCs aligned to five behaviors events (red curves). Gray curves indicate the Ca^2+^ responses of the individual mouse. H) Trial‐averaged Ca^2+^ activity during uphill, downhill, flat walking, and quiescent periods in 839 PCs from 6 mice. I) Percentages of uphill, downhill, flat walking, and quiescent neurons in all recorded mice (839 PCs from 6 mice). Gray circle: individual mouse. *p < 0.01; ***p < 0.001 (one‐way ANOVA followed by paired Student's *t*‐test, *F* = 168.78, *p* < 0.001).

As PCs receive inhibitory inputs from MLIs, we speculated that neuronal activity in these cells would be suppressed during ramp locomotion. To test this hypothesis, we injected PCP2/L7‐GCaMP6 into the cerebellum of mice to specifically label PCs (**Figure** **8**A,B) and then imaged their activity during ramp locomotion. Similar to previous Ca^2+^ imaging studies,^[^ ^18^, ^19^ ^]^ the PCs were characterized by striped dendrites (Figure 8C, Upper). We found that most PCs exhibited persisting discharge during the quiescent period and suppressed activity during locomotion (Figures 8C Lower, 8D–F). PCs' activity was negatively correlated with the movement speed both at the population and single neuron levels (Figure 8F). Moreover, most PCs showed decreased neuronal activity during ramp walking after the activity was aligned to different event markers (Figure 8G). Statistically, about 80% of PCs displayed stronger activation during the quiescent period (Figure 8H,I). To further examine whether PC activity is correlated with mouse position or moving direction, we compared PCs's activity when the mice ran in left or right directions. We found that PCs exhibited active activity when the mouse walked toward the slope and stopped firing after the mouse crossed the peak of the slope regardless of whether the mouse initiated locomotion from the left or right (Figure S7, Supporting Information). Further analysis showed that PCs displayed symmetrical activation patterns on both sides of the triangular ramp when the neuronal activity of the left trials was subtracted from the neuronal activity of the right trials (Figure S7B, Supporting Information), suggesting that PCs may represent ramp walking or the ramp environment at the population level.

### 2.7 MLIs and PCs Activity were Counter‐Balanced During Ramp Locomotion

To further examine the neuronal activity of MLIs and PCs simultaneously during ramping walking, we labeled the superficial layer of the cerebellum with AAV‐CaMKII*α*‐GCaMP6. Because the premotor of CaMKII was reported both in MLIs and PCs,^[^ ^18^, ^43^ ^]^ this method infected both MLIs and PCs (Figure S8A, Supporting Information). Similar to our cell‐type‐specific labeling method (Figure 8), PCs had striped dendrites (Video S2, Supporting Information), whereas the MLIs had smaller cell bodies, which can be distinguished during Ca^2+^ imaging with a miniscope (Video S3, Supporting Information). Because the morphology of individual neurons captured by PCA‐ICA algorithms was artificially confirmed to be neurons according to shape and size (details in Section 4), we were able to separate them into MLIs and PCs (Figure S8B, Supporting Information). Moreover, in contrast to MLIs, PCs displayed a higher event frequency and intensity (Figure S8C–E, Supporting Information). In this way, we simultaneously obtained the Ca^2+^ activity of MLIs and PCs during ramp locomotion. We found that MLIs specifically and persistently activated during ramp locomotion (Figure S9A,C, Supporting Information), especially on uphill or downhill phases (Figure S9B,C, Supporting Information), similar to the activity of MLIs labeled by syn‐GCaMP6 (Figures 2, 3, 4, 5). Furthermore, we found that a similar proportion of MLIs showed increased activation during uphill and downhill walking behaviors to that of syn‐GCaMP6 labeled mice (Figure S9B,D, Supporting Information). The PCs exhibited lower activity during ramp locomotion (Figure S10A, Supporting Information) and higher activity during the quiescent period (Figure S10B–D, Supporting Information). These results further confirmed that MLIs and PCs activity were counter‐balanced activated during ramp locomotion.

### 2.8 The Vermis is Required for Ramp Walking

We tested the running behaviors of mice in the L maze after inactivating the vermis and found that the mice stopped running after a few trials (data not shown). To dissect the function of the cerebellum in ramp walking, we trained the mice to perform forced running behavior on a treadmill (Figure S11C, Supporting Information). To do so, the inhibitory DREADD human Gi‐coupled M4 muscarinic receptor (AAV‐hM4Di) was expressed in lobule V of the cerebellar vermis (Figure S11A,B, Supporting Information) which was inactivated by clozapine‐*n*‐oxide (CNO, 10 mg kg^−1^, i.p.) 3 weeks after virus injection (Figure S11A, Supporting Information). Then the mice were required to run on a treadmill at the same speed (10 m min^−1^) for 8 min under uphill (20°), downhill (−20°), or flat (0°) conditions. For animal welfare, the tasks were stopped when the animals stopped running for 30 s. We found that mice injected with the control virus (AAV‐mCherry) or the mice with hM4Di expression after vehicle injection (saline, 10 mg kg^−1^, i.p.) showed normal running behavior under all three conditions (Figure S11C, Supporting Information). Interestingly, most mice with hM4Di expression and CNO injection stopped running under the uphill condition 3–4 min after the task started (Figure S11C, Supporting Information). In contrast, the same mice kept running for 8 min under downhill or flat conditions (Figure S11C, Supporting Information). These results indicate that ramp walking behavior was impaired after the vermis was inactivated, confirming that the lobule V of vermis is required for ramp walking, especially crucial for uphill condition.

To further confirm whether MLI activity is correlated with ramp locomotion, we also imaged MLIs activity when a mouse was walking on a treadmill. We found that MLIs also displayed higher activity during the uphill and downhill locomotion than during flat walking or quiescence (resting time for the incline switch) in a small box (Figure S11D,E–G). Furthermore, similar to walking on the triangular ramp, more than 60% of MLIs showed the highest activity during uphill or downhill walking on the treadmill (Figure S11H, Supporting Information). Thus, the activity of vermal MLIs was also specifically elevated during uphill and downhill locomotion on the treadmill.

## 3 Discussion

In the current study, by imaging the neuronal activity of MLIs and PCs in the vermis while mice were performing uphill and downhill walking behavior, we found that MLIs were specifically activated during uphill and downhill walking (Figures 2, 3, 4, 5, 6, 7), exhibiting persistently high activity during uphill or downhill phases on the slopes both during the freely‐moving condition (Figure 4) and forced‐moving condition (Figure S11, Supporting Information). Furthermore, MLIs encode the inclines of the slope (Figures 6 and 7) in different manners. In contrast, PCs exhibited decreased neural activity during ramp walking (Figure 8) and may represent the ramp walking or ramp environment at the population level (Figures S7,S10, Supporting Information and Figure 8). These results provide direct evidence that superficial MLIs provide timely predictive error signals during ramp walking to inhibit PC activity for adjusting locomotion behaviors.

Previous two‐photon imaging studies in head‐fixed mice have revealed that the cerebellum plays important roles in bruxing,^[^ ^16^ ^]^ the eye‐blink reflex,^[^ ^5^ ^]^ forelimb movement,^[^ ^20^ ^]^ and sugar‐water reward.^[^ ^39^ ^]^ However, studies carried out on immobilized animals restrict our understanding of how the cerebellum actively engages in many other behaviors, such as spatial navigation in 3D terrain, fighting, social interaction, and cognition, all of which require the study of freely‐moving animals and population‐scale imaging. Furthermore, neurons in the cerebellum showed decreased activity during self‐motion compared with passive movement.^[^ ^11^ ^]^ Although miniature microscope imaging has been widely used in deep brain regions^[^ ^44^, ^45^ ^]^ and the cerebral cortex,^[^ ^46^ ^]^ it is not commonly used for imaging the cerebellum in freely‐moving animals,^[^ ^33^, ^34^ ^]^ especially rarely for MLIs. In the current study, we developed a miniature microscope imaging technique that allowed us to successfully visualize the neuronal activity in the superficial layer of the cerebellum, especially in MLIs and PCs under a variety of freely‐moving behavioral paradigms, which will advance studies dissecting the circuit and function of the cerebellum in both motor and non‐motor related behaviors.

Locomotion has been extensively studied, both for gross^[^ ^25^ ^]^ and fine motor control.^[^ ^28^ ^]^ Unlike ambulation on a flat surface, locomotion on ramps includes both horizontal and vertical movement which requires dynamic estimation of gravity to adjust behaviors. Previous electrophysiological recording studies in non‐human primates and rodents have revealed the critical role of cerebellar neurons in controlling locomotion,^[^ ^24^ ^]^ anti‐gravity,^[^ ^11^ ^]^ and motor preparation,^[^ ^47^ ^]^ which indicated that the cerebellum may be crucial for motor behaviors on a ramp. By applying Ca^2+^ imaging to the superficial layer of the cerebellum at large‐scale and single‐cell resolution in freely‐moving mice, we found, for the first time, MLIs are involved in flat, uphill, and downhill walking both on triangle slopes (Figure 1, 2, 3, 4, 5, 6, 7) and the slopes on the treadmill (Figure S11, Supporting Information), and PCs represented the ramp walking and ramp environment at a population level (Figure 8, Figures S7 and S10, Supporting Information). In addition, previous studies have found that inactivation of the medial cerebellum did not influence the step adaptation of mice on a flat split‐belt treadmill.^[^ ^48^ ^]^ Consistently, we found that inactivation of the lobule V of vermal cerebellum specifically impaired the uphill locomotion but not flat ambulation (Figure S11, Supporting Information). These results provide direct evidence that the vermal cerebellum is important for ramp walking.

Previous studies have indicated that MLIs play an important role in cerebellar information processing by controlling PC activity via inhibitory synaptic transmission.^[^ ^4^ ^]^ MLIs were classed into an inner layer with basket cells and an outer layer with stellate cells,^[^ ^4^, ^49^ ^]^ which showed similar electrophysiological properties^[^ ^50^ ^]^ with irregular spontaneous activity.^[^ ^4^, ^49^, ^50^ ^]^ Recently, several studies have indicated that MLIs are activated during motor output.^[^ ^15^, ^35^, ^51^ ^]^ Moreover, in vivo studies indicated that MLIs contributed not only to sensorimotor information processing but also to precise motor coordination^[^ ^4^, ^52^ ^]^ and motor learning.^[^ ^53^ ^]^ And MLIs showed increased activity during locomotion compared to still conditions.^[^ ^15^, ^21^ ^]^ However, how MLIs are involved in locomotor behavior, especially on a ramp, was unclear. In the present study, we found synchronized neural activity in the vermis during ramp walking behaviors (Video S1, Supporting Information and Figure 1H), which echoes concerted neural activity previously recorded both from Crus II during licking behavior^[^ ^16^ ^]^ and the vermis during associative learning.^[^ ^17^ ^]^ Furthermore, mice experienced different kinds of movements in sequence in our behavioral paradigm (right trials: level, uphill, and downhill; left trials: uphill, downhill, and level). We found that different MLIs were synchronously activated at different phases of movement in sequence (Figure 5, Figure S6,S9, Video S1, Supporting Information). These results suggest that MLIs may timely modulate PC activity based on different locomotion stages.

Interestingly, although a larger percentage of MLIs showed increased response on uphill or downhill regardless of the size of slopes, some MLIs displayed diverse activity to the inclines of the slope (Figures 6 and 7). These results indicate that a larger part of MLIs play a key role in differentiating the slope size and some MLIs may generally identify the slope regardless of its steepness (similar response to different slope sizes) during uphill and downhill walking. More interestingly, we found some flat‐responsive neurons switched to ramp‐responsive neurons when the inclines of the slope increased. These results demonstrated that MLIs may serve for the perception of inclines during locomotion on the ramp. The different activation of MLIs at different uphill and downhill slopes and slope inclines may be correlated with the effort/calorie expenditure during locomotion. However, so far, we could not exclude the contribution of effort/calorie expenditure to the different representations of uphill and downhill walking in vermal cerebellum. In addition, there are few papers to study the neural coding mechanism of effort and calorie expenditure in the cerebellum. We may examine this possible mechanism in future studies.

Several studies in the last century have recorded the neural activity of climbing fiber,^[^ ^54^ ^]^ PCs^[^ ^23^, ^24^, ^55^, ^56^ ^]^ while a cat was walking on a treadmill. They found that PCs displayed step‐related activity,^[^ ^23^, ^24^ ^]^ and these activities rarely changed during uphill movements.^[^ ^24^ ^]^ These results indicate that Purkinje cells use phase‐locked discharging to synchronize with the step cycle of locomotion instead of the inclines of the slope. Consistent with the previous results, our results showed that PC activity was not specifically modulated by uphill or downhill locomotion (Figure 8 and Figure S10, Supporting Information). Thus, a question is raised: what's the role of MLIs if the PCs no longer encode the uphill or downhill during ramp walking? One possibility is that Purkinje cells encode error signals during walking, and re‐afferent sensory inputs are not much different for flat, uphill or downhill locomotion, so the error signals would be similar between different slopes. Previous studies also showed PCs in lobule V were activated by the stimulation of the ipsilateral forelimb^[^ ^23^ ^]^ and received somatosensory^[^ ^57^ ^]^ and cutaneous^[^ ^58^ ^]^ projections from the forelimb. Meanwhile, we did not find the different activities of PCs between ramp walking and level walking. So, PCs may have similar somatosensory inputs from forelimbs during ramp and flat locomotion. Furthermore, our results showed PCs displayed a dichotomous activation pattern along the peak of the slope, which may represent the ramp walking or ramp environment at the population level, but do not modulate the locomotion on the ramp timely (Figure S7, Supporting Information).

**Figure 9:**
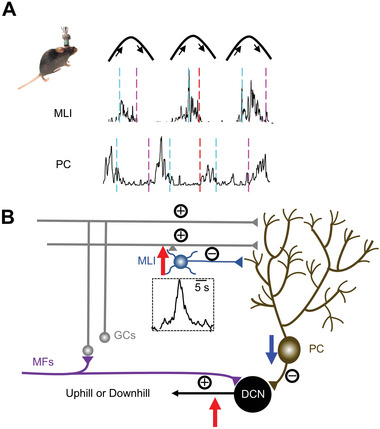
Working model to explain the micro‐circuit in the vermal cerebellum during ramp walking. During uphill or downhill locomotion, different kinds of cutaneous, somatosensory, and locomotor information will enter the cerebellum through mossy fiber. The excitatory information from mossy fiber transfers to GCs and deep cerebellar nuclei (DCN). MLIs directly receive inputs from GCs and then timely represent different kinds of locomotion. PCs integrate the excitatory inputs from GCs and the inhibitory inputs from MLIs which result in anti‐correlated with the locomotion on the slope and send out less inhibitory signals to DCN. DCN could compare the inputs from MFs and the integrated inhibitory signals from PCs. Because of the decreased activity of PCs during ramp walking, DCN will send out more excitatory activity to modulate locomotion on a slope. DCN, deep cerebellar nuclei; GCs, granule cells; PCs, Purkinje cells; PFs, parallel fibers; MFs, mossy fibers.

Together with the neural activity of MLIs and PCs in our Ca^2+^ imaging studies and previous electrophysiology recording results,^[^ ^15^ ^]^ we proposed a working model to explain the micro‐circuit in the vermal cerebellum during ramp walking (**Figure** **9**). During uphill or downhill walking, different kinds of cutaneous, somatosensory, and locomotion information will be transmitted to the cerebellum through mossy fiber. The excitatory information from mossy fiber transfers to GCs and deep cerebellar nuclei (DCN). MLIs directly receive input from GCs and then timely represent different kinds of locomotion. PCs integrate both the excitatory inputs from GCs and the inhibitory inputs from MLIs, which result in decreasing response during the ambulation on the slope and send less inhibitory signals to DCN. DCN could compare the input from MFs and the integrated inhibitory signals from PCs. Because of the decreased inhibitory activity from PCs, DCN will send out more excitatory activity to modulate locomotion on a slope. Taken together, our results dissected a micro‐circuit in the vermal cerebellum for regulating ambulatory behavior in a 3D terrain.

## 4 Experimental Section

### Subjects

Adult male C57BL/6 mice (8‐10 weeks, 20–25 g) were used for all experiments. The mice were housed 4 or 5 per cage at room temperature (25 ± 1 °C) and humidity (40 ± 5%) on a 12 h light/dark cycle (lights on from 07:00 AM to 07:00 PM) with food and water ad libitum until surgery. All behavioral tests were carried out in the daytime. All experiments were conducted in accordance with the guidelines for the care and use of laboratory animals at Zhejiang University and were reviewed and approved by the Animal Advisory Committee at Zhejiang University (ZJU20210138).

### Ramp Locomotor Behavior

As one of the common forms of movement, uphill and downhill walking in hiking were often used to study energy consumption.^[^ ^29^, ^30^ ^]^ To better understand the neural mechanism of uphill and downhill movement, an uphill and downhill paradigm was designed, in which an unbalanced L maze that included a longer horizontal arm (80 cm) and a shorter vertical arm (40 cm) was used to increase the comparison. As shown in Figure 2A and Figure S1B, Supporting Information, an isosceles triangle ramp (base angle: 45°, length: 48 cm, height: 24 cm) was placed in the horizontal arm of the L maze, and food pellets were alternately placed 1 cm away from the ends of the two arms. In this task, mice ran backward and forward to receive rewards manually delivered by trainers. Based on the reward locations, the trials were divided into right trials (food pellets at the end of the horizontal arm) and left trials (food pellets at the end of the vertical arm). With this method, the locations of uphill and downhill were opposite in the two kinds of trials, thus excluding the effect of running direction and ramp location. The detailed experimental procedure is shown in Figure 1A. To accelerate the experimental procedure, the mice were restricted to 80% daily food intake 14 days after cranial surgery and they were weighed every day to maintain no less than 85% weight compared to the same mice before food restriction. Then, the mice went through 4 days of pre‐training with a dummy miniscope on their heads in the L maze to avoid the effects of stress and novelty during later behavior and imaging. During behavioral training and Ca^2+^ imaging, behavioral videos were captured by a high‐definition camera at 30 frames s^−1^, reaching a maximum of 35 trials within 15 min. Incomplete trials in which the mouse turned back in the middle of the L maze were excluded from the analysis.

Because of the camera wire and human interference in the task, the animal's movement in the L maze was tracked by a customized algorithm based on deep learning and a background subtraction algorithm—DeepBhvTracking.^[^ ^59^ ^]^ To quantify the behavior, walking was divided into three groups based on the movement states and location in the L maze: 1) Uphill, defined by climbing up the ramp; 2) Downhill, defined by climbing down the ramp; 3) Flat walking. To avoid any other influence on flat walking, it was only measured in the vertical arm.

To further analyze mouse behaviors, five event markers were both manually selected based on the animal's location and movement state and automatically identified by the movement trace (over 0.3 m s^−1^ identified as locomotion). 1) Trial start: in this task, mice usually consume the food pellet and turn around for another trial. The trial start was defined by the beginning of visible movements (also confirmed by the velocity, over 0.3 m s^−1^) after turning around; 2) Upstart: the animal began to climb up the ramp; 3) Peak: the center of the mouse was on the peak of the ramp; 4) Down end: a hind limb began to leave the ramp; 5) Reward: the mouse reached and began to consume the food pellet.

### Surgery

Cranial window surgery was performed based on previous methods with some modifications.^[^ ^28^, ^32^ ^]^ Briefly, the stereotaxic apparatus and surgical instruments were carefully sterilized with 70% ethanol 30 min before surgery. Then, the mice were anesthetized with sodium pentobarbital (0.15 mL g^−1^, i.p.) and pre‐treated with dexamethasone (0.2 mg kg^−1^, i.p.) to relieve surgery‐related edema and inflammation. The fur between the eyes and ears was trimmed and the skin was disinfected with three alternated swabs of 70% ethanol. To alleviate acute pain during surgery, 2% lidocaine was used locally (s.c.). An incision of ≈4 mm was made over the cerebellum, and the surface was exposed. To prevent tissue growth and to stabilize the coverslip, the periosteum was scrapped off with a scalpel blade and the skull was cleaned and dried with 100% alcohol, especially around the target craniotomy region. Most importantly, a piece of neck muscle in the vertical position was removed to stabilize the coverslip and baseplate. A high‐speed micro‐drill (typically between 7000 and 10 000 rpm) with a 0.5 mm burr was used to create a circular craniotomy (slightly >3 mm) around the desired viral injection location (1.66 mm caudal to lambda).

### The Infusion of GCaMP6 into the Superficial Layer of Vermis

Viruses were infused into the surface of the cerebellum based on previous methods with some modifications.^[^ ^28^, ^32^, ^46^ ^]^ To better visualize the activity of superficial cerebellar neurons with the combined cranial window and miniature microscope technique, superficial labeling was required to match the focal plane of the microscope and to avoid deep light contamination. Based on the previous method,^[^ ^32^ ^]^ GCaMP6 was infused into the superficial layer of the cerebellum by a glass pipette with a tip size of ≈0.3 mm. The details of infusion were as follows: 1) the bone debris around the edge of the window was carefully removed to avoid bleeding; 2) the desired imaging region was localized and large blood vessels that may induce vibration of the video during imaging were avoided; 3) a small piece of dura around the imaging region was removed; 4) glass pipette with tip size 0.3–0.5 mm was filled with AAV2/9‐syn‐GCaMP6s(0.6 µL per animal, Shanghai Sunbio Medical Biotechnology, China); 5) the pipette was positioned on the cerebellar surface and lowered 0.4–0.6 mm; 6) with a micro‐pump, the virus was infused into the brain at 60 nL min^−1^; 7) 5 min after the infusion, a sterilized 3.5‐mm glass coverslip (Thomas Scientific, Catalog # 64–0720, 0.15 mm thick) was affixed to the craniotomy at the same level as the surrounding skull; 8) the scalp was sutured for improved postoperative recovery; 9) after 14–21 days, the baseplate of a miniaturized integrated fluorescent microscope (Inscopix) was affixed above the labeled region (Figure 1D). With this method, the virus mainly labeled the superficial layer of the cerebellum, and was the key step for successful imaging with a one‐photon microscope. To specifically label the PCs, AAV2/8‐PCP2/L7‐GCaMP6f was used based on the previous report.^[^ ^18^ ^]^ To image the PCs and MLIs at the same time, AAV2/9‐CaMKIIa‐GCaMP6s was infused to the surface of the vermal cerebellum.

### Calcium Imaging in Behaving Mice

Ca^2+^ imaging was performed when animals were walking on a slope via the head‐attached microscope (Inscopix, version nVista 2.0; LED power: 0.6–1.0 mW; camera resolution: 1440 × 1080 pixels). Images were acquired at 30 Hz using nVista HD software (Inscopix, version 2.1). Before each imaging session, dust on the coverslip and baseplate was carefully removed with a piece of lens paper and the microscope was attached. The imaging field of view was ≈900 × 650 µm^2^ at 0.65 mm per pixel resolution. The imaging depth was determined by adjusting the focus of the microscope until clear neuronal signals were observed, appearing as flashing spots in the videos. The cerebellum is characterized by its regular 3‐layer organization: the molecular, Purkinje, and granule cell layers. The neurons in each layer showed distinct density and cell size. With increasing imaging depth, blood vessels were clearly visualized first together with sparse small inhibitory interneurons (5–7 µm in diameter, 0–100 µm below the pial surface); this was the molecular layer. Then, a few large Purkinje cells (20 µm in diameter, 100–120 µm below the pial surface) were seen. Notably, the granule cell layer with single‐cell resolution could not be reached in most images with a miniature microscope. During imaging, the behavioral videos were recorded by a high‐definition camera (1080p, Ordro, 282), which was synchronized with Ca^2+^ imaging using the trigger‐out signal from nVista HD.

### Extraction of Neuronal Ca^2+^ Signals

Ca^2+^ imaging videos were analyzed using the Imaging Process Toolbox (Inscopix) and custom‐written scripts in MATLAB following published algorithms and based on previous publications.^[^ ^28^, ^32^ ^]^ Raw videos were first down‐sampled four‐fold along spatial dimensions to reduce file size and noise and were corrected the motion with the stabilizer plugin in Image J (NIH), Imaging Data Processing (Inscopix), or NoRMcorre toolbox.^[^ ^60^ ^]^ The mean fluorescence intensity of each pixel during a recording session (≈15 min) was calculated as *F*_0_ and changes in pixel intensity at time t were expressed as (*F*_t_ – *F*_0_)/*F*_0_ or ∆*F*/*F*_0_. To extract active neuronal Ca^2+^ signals, principal component and independent component analysis (PCA‐ICA) was applied to analyze the spatio‐temporal data matrices of Δ*F*/*F*_0_ using the CellSort and fastICA toolboxes (these toolboxes are freely downloadable from MATLAB Central). This analysis decomposed a spatiotemporal data matrix into independent components based on the skewness of data distribution. Each component had a characteristic spatial filter over the imaged area and a corresponding temporal signal during the imaging period. The spatial filter and the temporal signal of each component were graphed and inspected by investigators who were blind to the experimental conditions of each video. If the spatial filter for a component overlapped with the dark shadows cast by blood vessels in the F_0_ image, this component was likely contributed by blood flow and was therefore rejected. Moreover, if the spatial filter for a component displayed a larger soma or stippled morphology, this component was a Purkinje cell and rejected. Last, because Ca^2+^ signals show a characteristic fast‐rising and slow‐decaying time course, the temporal skewness of Ca^2+^ signals were expected to be positive and those components with skewness <1 were rejected. For each selected component, the location of the MLI was identified as the brightest spot (3 × 3 pixels) of the spatial filter. The corresponding temporal signal of an MLI was calculated from the Δ*F*/*F*_0_ video by subtracting the median value of the background area (outside the cell body) from the average value of the MLI area.

PCs were found to display bigger somata or striped dendrites parallel to the body axis both in two‐photon and miniscope imaging studies.^[^ ^18^, ^33^, ^34^ ^]^ Although most dendrites' signals come from the backpropagation of soma, the neural activity between soma and dendrites may be different. So, in the authors' studies, the dendrites' activity of PCs was only analyzed. After the authors got the spatial filters corresponding to PCs through PCA/ICA, the spatial filters were truncated by setting the weights of all pixels with values less than 2 s.d. to zero, above the mean pixel weight. These truncated spatial filters were applied to the Δ*F*/*F*_0_ videos to extract fluorescence traces for each Purkinje neuron.^[^ ^18^ ^]^

To identify periods of increased neuronal activity, the rising phase of each Ca^2+^ event (peak ΔF/F_0_ >3 standard deviations of baseline fluctuation) was searched for, which is closely associated with neuronal spiking activity. The start of this rising phase was detected when the 1st derivative of Δ*F*/*F*_0_ (calculated in a 200‐ms moving window) rose above 0 and continued to increase above five standard deviations of baseline fluctuation, and the end of this rising phase was detected when the 1st derivative of Δ*F*/*F*_0_ fell below 0.

### Data analysis

To visualize the activity patterns of imaged neurons during the uphill and downhill movement, the active event traces of each neuron were aligned with the five previously‐defined behavioral actions: “Trial start”, “Up start”, “Peak”, “Downhill end” and “Reward”, then averaged across trials. The resulting traces from all recorded neurons were sorted based on their peak activation time during the task and displayed in temporal raster plots (Figure 2). Clearly, a large proportion of MLIs was uphill‐ or downhill‐related. To analyze the correlation between mice velocity and neuronal activity, the Pearson's correlation coefficient between the neuronal activity of recorded neurons and the speed at trial start, upstart, peak, down end and reward were calculated.

To determine the walking‐related neurons, 2 s of baseline activity with low movement (the speed is <0.3m min^−1^, usually the period after food consumption and turning around) was chosen. The mice kept still on the flat surface. Then the activity in different windows was compared with baseline activity (test‐test, *p* < 0.05). The uphill neurons were defined by their significantly higher activity than baseline and downhill; it was the highest among all other locations in the L maze. The downhill and flat walking neurons were defined by similar methods and showed the highest activity in the downhill location or flat walking region, respectively.

To systematically analyze the walking‐related neurons of all recorded neurons, the locations in the L maze were divided into 130 bins (bin size:1 cm, 90 for the horizontal arm, and 40 for the vertical arm). The activity differences between the right and left trials of all the neurons were projected into the 130 bins (average based on mouse location) and then sorted based on the location of peak activation. Based on the location and animal's location, the L maze was divided into eight windows: from w1 to w8; in right trials, “w5” represented uphill and “w6” represented downhill, and the opposite in left trials. Clearly, a larger group of MLIs showed positive activity in w5 and negative activity in w6 indicating uphill‐specific neurons. Another group was downhill neurons.

To identify the phase‐specific neurons, the activity of individual neurons was aligned to the peak of slope and averaged across trials (10‐s analyzed window). Then, the standard deviation of trial‐averaged activity was used as the threshold to define the MLIs' activation time. If the activation time was longer than 2 s (2/3 of average uphill duration) and the activated window was located before the slope peak, the neuron was defined as an uphill neuron. If the activation time was longer than 2 s and the activated window was located behind the slope peak, the neuron was defined as a downhill neuron. If activation was found both before and after the peak of slope, the neuron was defined as both neurons. If the activation time was <1 s and close to peak, the neuron was defined as a peak phase‐change neuron. To identify the phase‐change upstart neurons, the activity of each neuron was aligned to the start point of uphill and averaged across trials, the standard deviation of trial‐averaged activity was also used as the threshold to define the MLIs activation time. If the activation time was <1 s and the activated window was just before the start point, the neuron was defined as a phase‐change pre‐uphill neuron. If the activation time was <1 s and just after uphill movement, the neuron was defined as a peak phase‐change post‐uphill neuron. To identify the phase‐change downhill neurons, the activity of individual neurons was aligned to the end of downhill. If the activation time was <1 s and close to the end of downhill, the neuron was defined as a phase‐change post‐downhill neuron.

### Decoding Uphill and Downhill Movement

To classify the neuronal signals and further predict the behaviors, the support vector machine (SVM) algorithm was used and an SVM classifier was trained for each mouse.^[^ ^42^ ^]^ The classification was performed using the LIBSVM library (https://www.csie.ntu.edu.tw/~cjlin/libsvm) and scripts run on MATLAB R2020a.

During each trial, the behavior and the corresponding MLI Ca^2+^ signal were recorded at 30 frames s^−1^, and the behaviors in each frame were manually labeled into four types: uphill, downhill, flat walking, and others. The Ca^2+^ signals were normalized to the [0, 1] range for further analysis. For each mouse, different trials were randomly allocated into two sets: a training set and a test set. The numbers of trials in the two sets were relatively equal. Then the SVM classifier was trained with the training set, and the trained classifier was used to classify the test set. During training, the SVM parameters were adjusted for better performance. ten‐times cross‐validation was used to find the best parameters.

After classification, a predicted label for each frame was obtained in different trials. The accuracy was calculated as follows: frames labeled correctly by SVM/manually labeled frames. To compare the prediction accuracy with chance, the Ca^2+^ signals of the test set were shuffled 1000 times, then classified with a trained classifier.

### The Expression of hM4Di in the Vermis

Adult mice (6‐8 weeks old) were deeply anesthetized with sodium pentobarbital (0.15 mL g^−1^, i.p.) before surgery. In addition, 2% lidocaine was given subcutaneously before surgery. Intracranial injections were conducted with a stereotaxic frame. The skull was exposed, and two small holes (1 mm diameter, both sides) were drilled using a high‐speed microdrill. Four hundred nanoliters of viruses (AAV‐hSyn‐hM4D(Gi)‐mcherry or AAV‐hSyn‐mCherry, Shanghai Sunbio Medical Biotechnology, China) were injected into the lobule V of the vermis (AP: 1.66 mm, ML: 0.0 mm, DV: 1.0 mm) using pulled and beveled glass pipettes at a flow rate of 30 nL min^−1^ controlled by a microcontroller. The expressions of hM4Di and mCherry virus were checked after behavioral testing and mice with only lobule V expressed were used for analysis.

### Running Test on a Treadmill

The locomotion of mice in the L maze after inactivation of the vermis was tested and it was found that mice stopped running after a few trials. So, to dissect the function of the cerebellum in ramp walking, the locomotion of mice was tested on a treadmill at different inclines (ZH‐PT/5S; Anhui Zhenghua Co., Ltd., China). To train the locomotion of the mice, the speed of the treadmill was set to the average velocity in the L maze (10 m min^−1^). Then, the mice were put in the middle of the treadmill and ran following the movement of the treadmill. Sometimes, the mice fall into the back region (1/4) in the treadmill, and the mice will receive a foot shock (1.0 mA). If a mouse stopped running and stayed in the shock region for 30 s, the task was stopped.

In this experiment, two weeks after virus injection, mice were handled and habituated to a variable‐angle of the motor‐powered treadmill for four consecutive days before the treadmill test.^[^ ^61^ ^]^ Usually, after four consecutive days of exercise, mice would skillfully run on the treadmill at different inclines (0°, 20°, and −20°). To inactivate the neurons in the vermis, CNO (10 mg kg^−1^) or saline (0.1 mL kg^−1^) was intraperitoneally injected for two consecutive days. 60–80 min after the injection of CNO or saline, running behavior at different angles was tested at a speed of 10 m min^−1^ (average speed of mice in the L maze). It was found that only the mice with hM4Di expression and CNO injected stopped running under the uphill condition 3–4 min after the task start, even when an electrical shock (1.0 mA) was delivered at the back of the treadmill. Mice were removed from the treadmill once they had stopped running for 30 s. During the Ca^2+^ imaging experiments, the mice ran on the treadmill at different inclines respectively (0°, 20°, and −20°) and stayed in a little box during angle transformation.

### Histology

The expression of GCaMP6s and hM4Di in cerebellar cortical cells was histologically confirmed in fixed brain tissue from the mice after experiments. Mice were anesthetized with pentobarbital and perfused with saline and 4% paraformaldehyde. Brains were post‐fixed with 4% paraformaldehyde for 24 h and then immersed in 15% and 30% sucrose. Sagittal sections were cut at 30 µm on a cryostat microtome (Leica CM1950, Germany). Next, the sections were mounted on the coverslips and scanned with a Leica SP8 confocal laser‐scanning microscope (Leica Microsystems, Germany) using 10× and 20× objectives, hybrid (HyD) detectors for sensitive detection, and sequential scan mode. As shown in Figures 1F and 8B, most labeled GCaMP‐positivize neurons were annulus, indicating that they were healthy.

### Statistics

Prior to data analysis, the outliers in each group were removed using the isoutlier MATLAB function, using the median method. Then, the imaging and behavior data were analyzed by one‐way or two‐way analysis of variance (ANOVA) followed by Bonferroni's test for multiple comparisons. The student's *t*‐test was used for two group comparisons. Error bars in all figures represented the mean ± SEM and the number (*n*) of samples was indicated in the legends. All the analyses and figures were performed using MATLAB in the present study.

## Conflict of Interest

The authors declare no conflict of interest.

## Author Contributions

L.X.J. and G.L.X. designed the experiments, L.C.F. applied behavioral tests and imaging, Y.C.C. performed decoding, S.G.L. tracked animal behavior, L.C.F., W.X.T., and Z.Y. performed histology, L.X.J., L.C.F., and G.L.X. analyzed the data and made the figures, L.X.J., G.L.X., and S.Y. wrote the paper.

## Supporting information

Supporting Information

Supplemental Video 1

Supplemental Video 2

Supplemental Video 3

## Data Availability

Although the Ca^2+^ imaging data files are large (more than 1TB), the original data that support the findings of this study are available upon reasonable request.
