# Supplementary material for: Deconstruction of Vermal Cerebellum in Ramp Locomotion in Mice
Source: Adv Sci (Weinh). 2022 Nov 14;10(1):2203665. doi: 10.1002/advs.202203665 (PMC9811470; doi:10.1002/advs.202203665)
Supplement: Supplementary file 1 — Supporting Information [file ADVS-10-2203665-s003.pdf]

ADVANCED SCIENCE
Open Access

Supporting Information

for *Adv. Sci.*, DOI 10.1002/advs.202203665

Deconstruction of Vermal Cerebellum in Ramp Locomotion in Mice

Chenfei Lyu, Chencen Yu, Guanglong Sun, Yue Zhao, Ruolan Cai, Hao Sun, Xintai Wang, Guoqiang Jia, Lingzhu Fan, Xi Chen, Lin Zhou, Ying Shen, Lixia Gao* and Xinjian Li**

Supplementary figures 1-11

Lyu et al. Figure S1

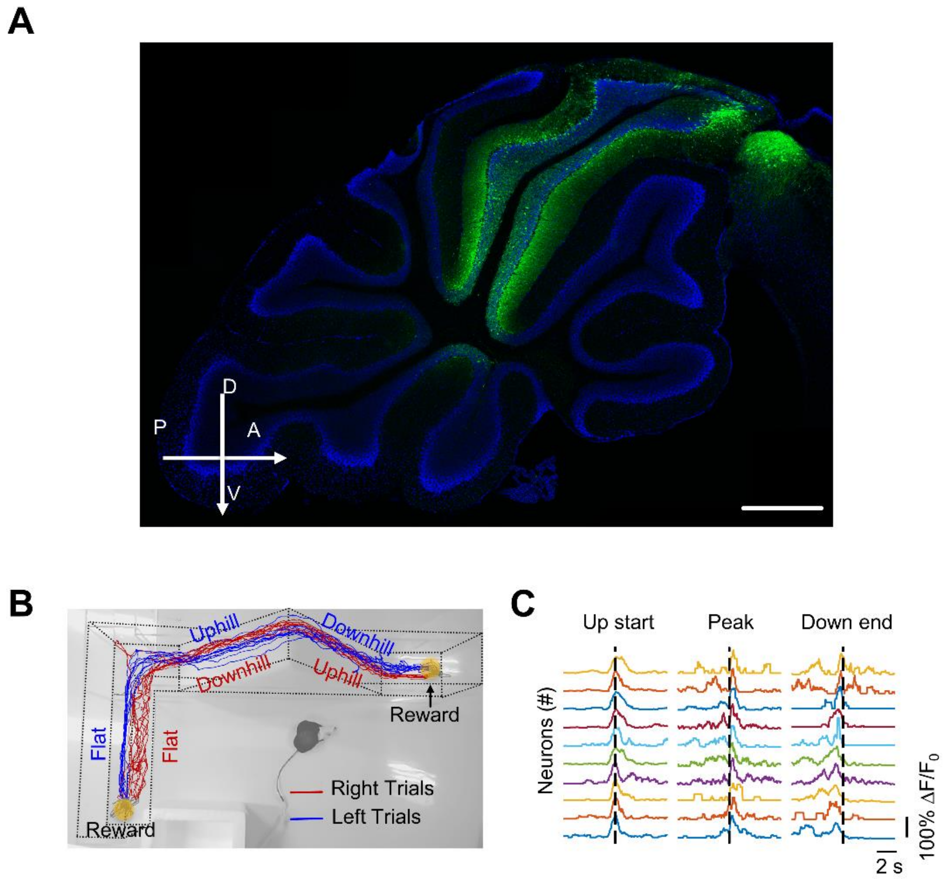

Figure S1. Representative neurons activated by ramp locomotion

(A) Image showing that the GCaMP is expressed in lobule V of the cerebellum (scale bar, 1 mm).

(B) Represent image showing the uphill and downhill locomotion of an animal on a ramp in the L maze with a 45°and 48 cm (height) triangle of Styrofoam in the middle of the horizontal arm. Mice are required to climb up and down the ramp to receive a food reward at either end of the arm (orange circles). The trials were divided into right (red) and left (blue) trials depending on the location of the reward. Each trial was divided into three movement phases: uphill, downhill, and flat walking. Red, movement traces of right trials; blue, left trials.

(C) Example fluorescent traces showing event-specific Ca^{2+} activity in the vermis. Left, aligned with ‘up start’; middle, aligned with ‘peak’; right, aligned with ‘down end’.

Lyu et al. Figure S2

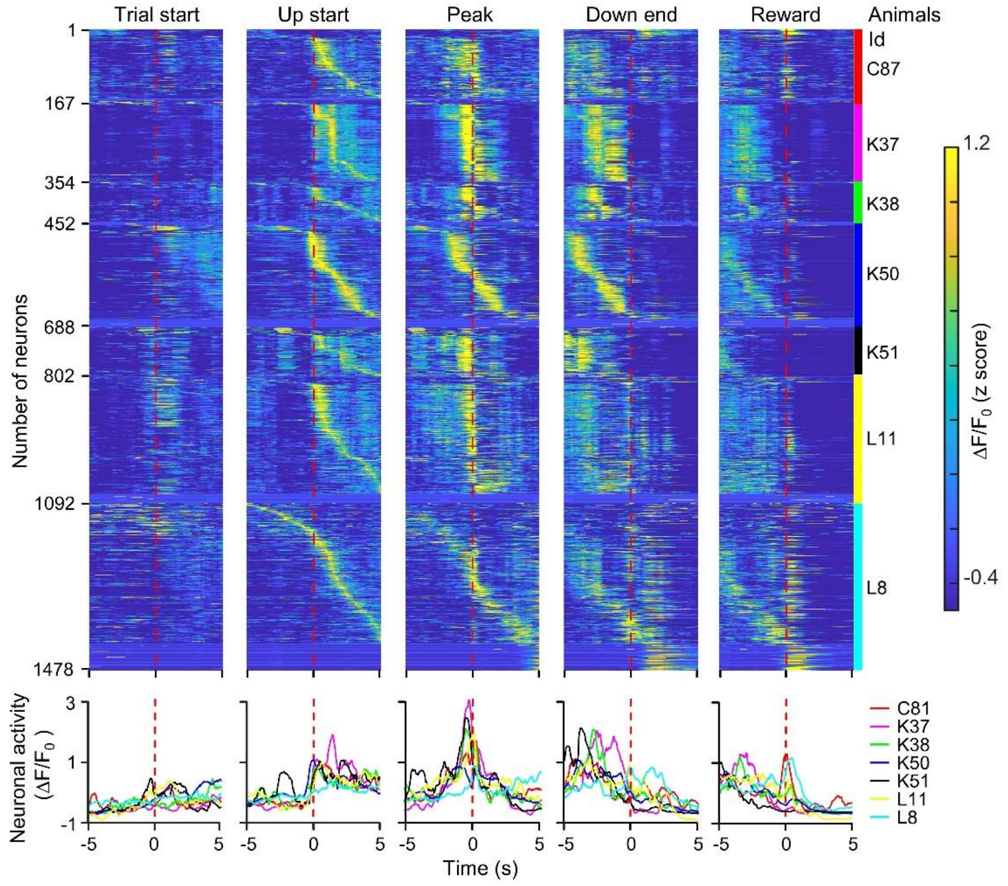

Fig. S2 Cerebellar vermis encodes ramp locomotion in each individual mouse

Upper, Raster plots showing event-specific Ca^{2+} activity during ramp walking in each recorded mouse. The different color in the right on the Y-axis represent different numbered mice. Bottom, the averaged Ca2+ responses of MLIs of invidual mouse were aligned by the 5 behaviors events.

Lyu et al. Figure S3

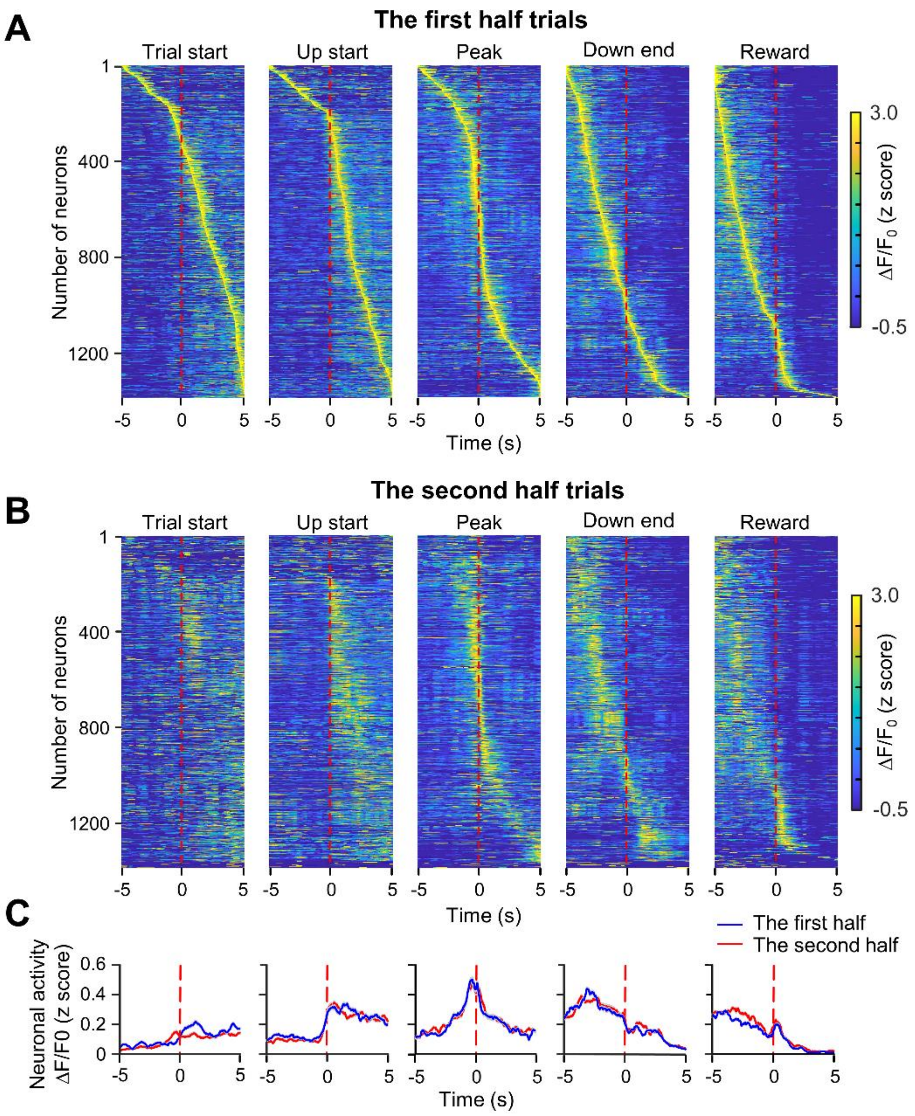

Figure S3. Cross-validation of MLIs activity during ramp locomotion

(A) Averaged neuronal activity of the first half trials aligned by different behavior events during ramp locomotion

(B) Averaged neuronal activity of the second half trials during ramp locomotion

(C) The comparison of the MLIs activity in the first half and second half trials. The activity of all MLIs was first averaged cross trials and then averaged across cells. Red: the first half trials; Blue: the second half trials.

Lyu et al. Figure S4

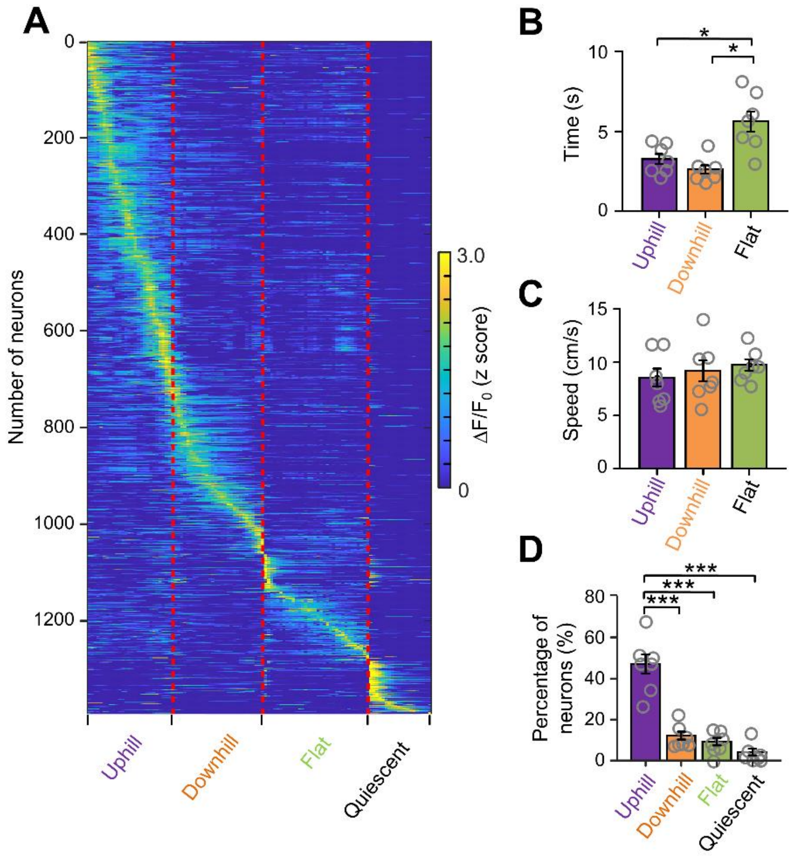

Figure S4. MLIs activity in the trials with similar running speed and duration between uphill and downhill walking

(A) Trial-averaged Ca^{2+} activity during uphill downhill, flat walking, and quiescence period of 1487 MLIs in 7 mice in the trials with similar running speed and time between uphill and downhill phase.

(B) The averaged running time (upper, one-way ANOVA, F=18.96, p<0.001) in the trials with similar running speed between uphill and downhill walking in 7 mice. *p <0.05 (one-way ANOVA followed by paired Student's t- test).

(C) The averaged running speed (lower, one-way ANOVA, F=2.2, p=0.14) in the trials with similar running speed trials between uphill and downhill walking in 7 mice. *p <0.05 (one-way ANOVA followed by paired Student's t- test).

(D) Proportion of uphill, downhill, flat walking, and quiescent neurons in the trials with similar running speed between uphill and downhill walking in 7 mice.

Lyu et al. Figure S5

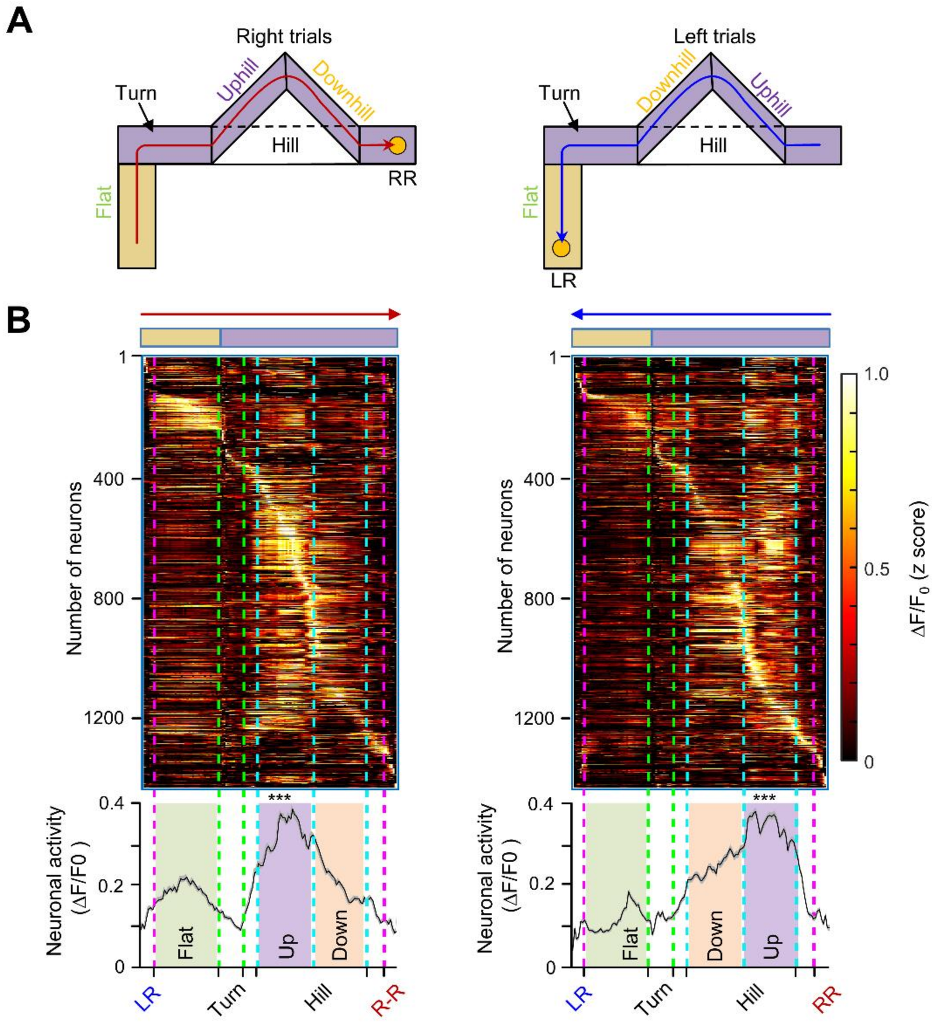

Figure S5. Populations of vermal MLIs respond to locomotion on a ramp

(A) Schematic showing event segments of ramp-walking in the right reward (red) and left reward trials (blue). Arrows indicate the movement directions.

(B) **Upper:** pseudo-color map showing MLIs activity in the L maze. The Ca^{2+} transients of all recorded neurons were projected to 130 bins in the L maze and then sorted based on the location with maximal Ca^{2+} activation in the L maze. Each row indicates the Ca^{2+} transient activity of a single cell. Red arrows, right trials; blue arrows, left trials; pink dashed line, reward location; the area between green dotted lines, location for turning; area bounded by cyan dotted lines, triangular ramp). **Lower**: averaged response of 1487 granule cells in the L maze. **Left column:** right trials; **right column**: left trials (***$p < 0.001$ between uphill and level walking, Student's *t-test*).

Lyu et al. Figure S6

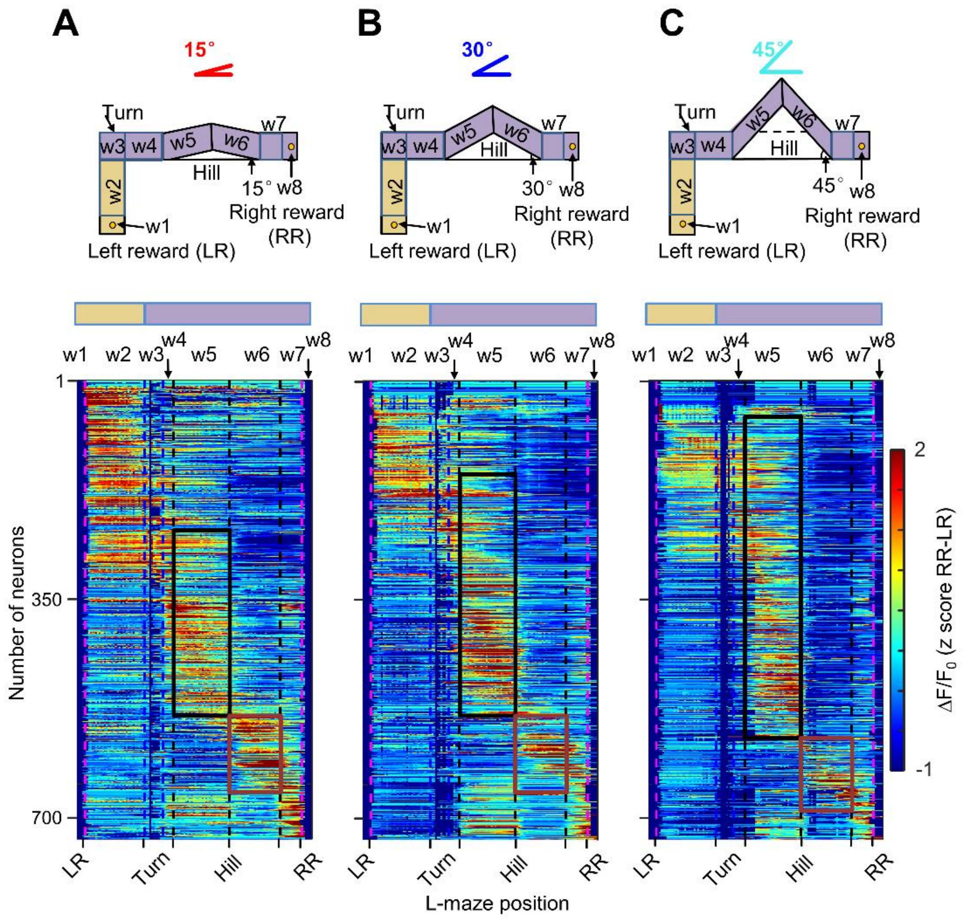

Figure S6. Pseudo-color map showing the subtraction of neuronal Ca^{2+} activity at each location between right and left trials.

(A) The activity difference between right and left trials on the 15° slope. The cells were sorted by the peak activity in different spatial windows.

(B) The activity difference between right and left trials on the 30° slope. The cells were sorted by cell sequence in different spatial windows.

(C) The activity difference between right and left trials on the 45° slope. The cells were sorted by cell sequence in different spatial windows.

Lyu et al. Figure S7

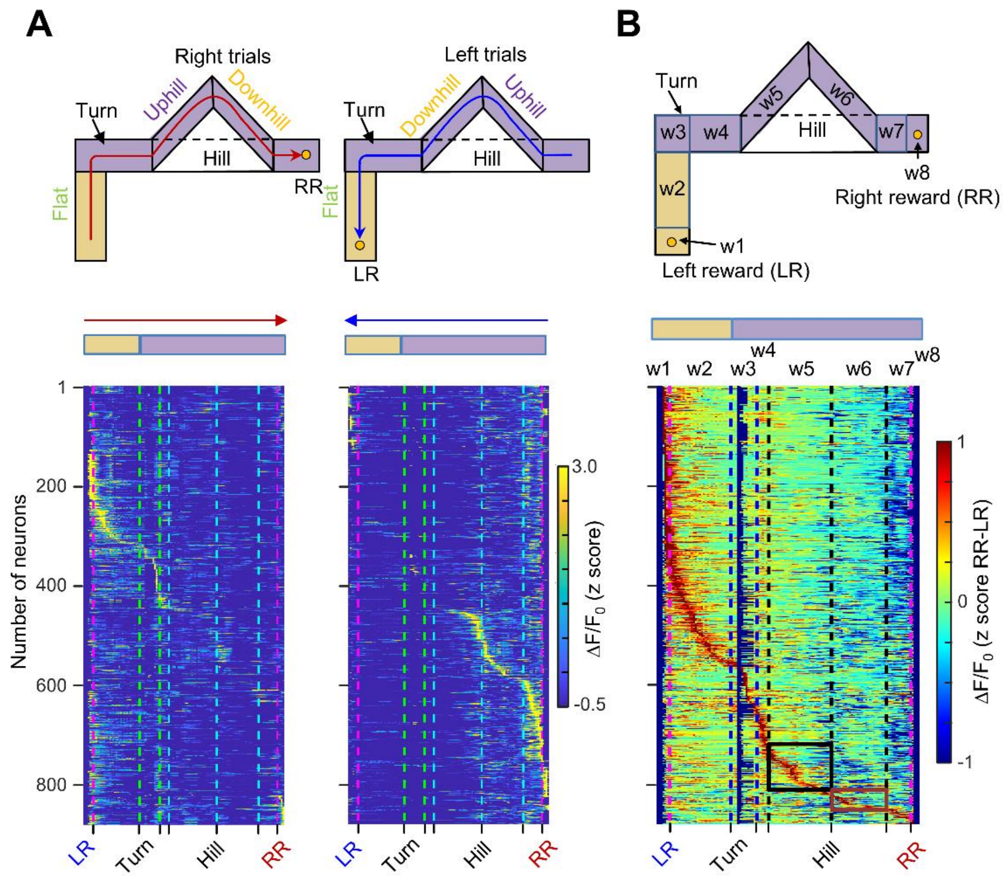

Figure S7. PCs displayed symmetry activity along the peak of the triangle slope at the population level

(A) Pseudo-color map showing PCs activity in the L maze. Left: PCs activity in right trials; Right: PCs activity in right trials (839 PCs from 6 mice).Pseudo-color map showing the subtraction of PCs activity at each location between right and left trials.

(B) Notably, there are few uphill and downhill PCs. PCs displayed symmetry activity along the peak of the triangle slope at the population level when subtracting the activity between the left and right trials, which indicates PCs may represent the ramp walking or ramp environment at the population level.

Lyu et al. Figure S8

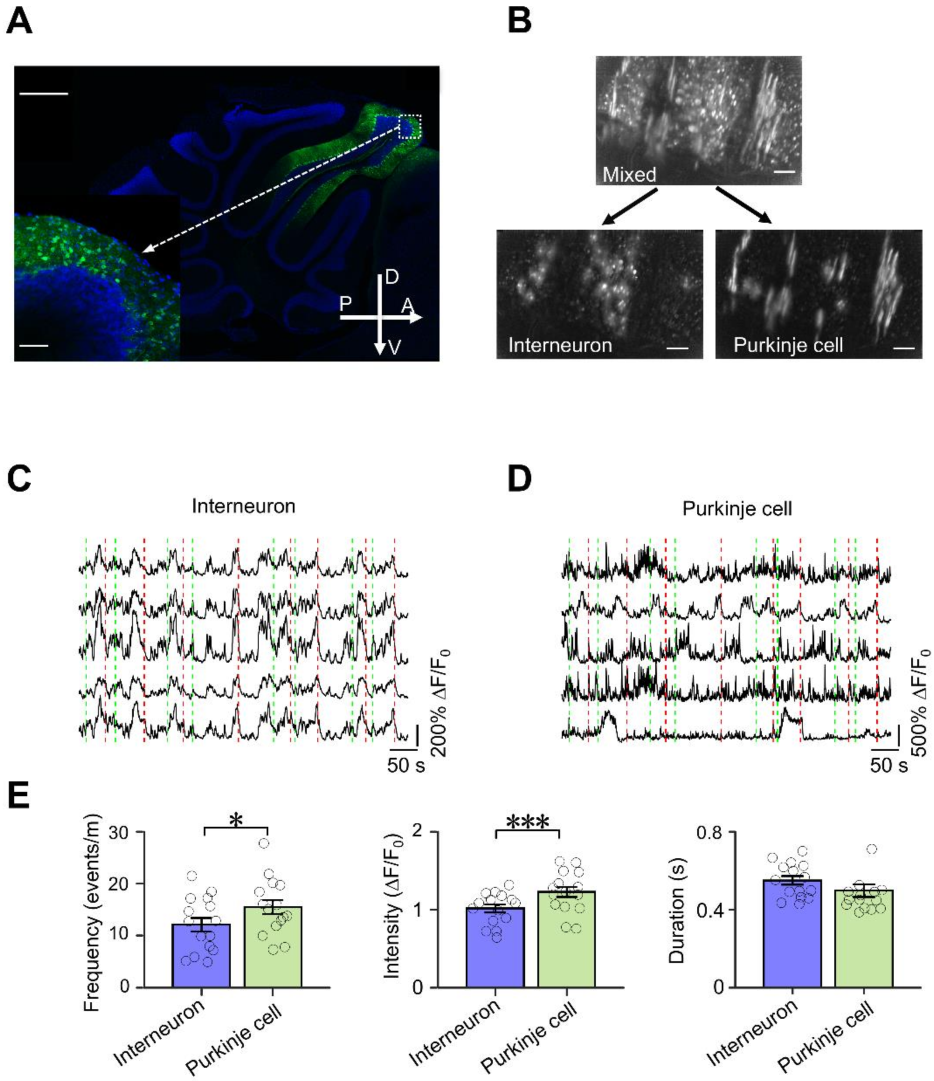

Figure S8. Imaging neuronal activity of MLIs and PCs together

(A) Image showing that GCaMP6 is expressed in the MLIs and PCs of lobule V under promotor of CaMK2α (scale bars, up: 500 μm; down: 50 μm).

(B) Upper: single-frame Ca^{2+} $\Delta F/F_0$ images showing mixed neurons labeled by -CaMKIIa-GCaMP6. Both MLIs and PCs were labeled. Lower: single-frame $\Delta F/F_0$ images showing interneurons (left) and Purkinje cells (right) separated from mixed imaging by PCA-ICA algorithms (scale bars, 100 μm)

(C) Example traces of Ca^{2+} transients of individual interneurons.

(D) Example traces showing the Ca^{2+} transients of individual Purkinje cells.

(E) Averaged Ca^{2+} event frequency (left), event intensity (middle), and event duration (right) in interneurons and Purkinje cells of 9 animals (**p <0.05; **p <0.01*, paired Student's *t-test*).

Lyu et al. Figure S9

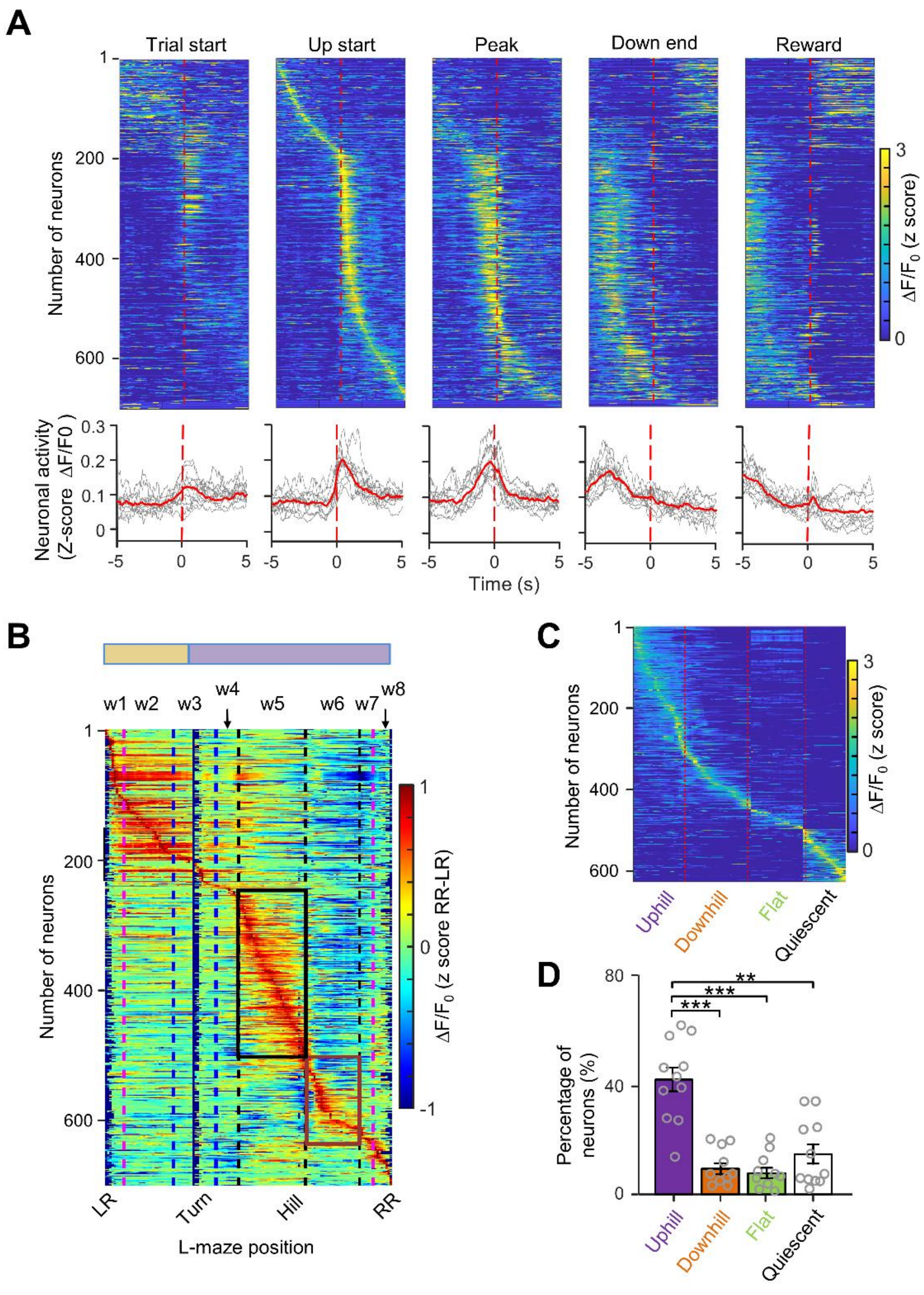

Figure S9. MLIs activity was elevated during uphill and downhill locomotion when imaged together with PCs

(A) Upper: trial-averaged MLIs separated from-CaMKIIa–GCaMP6-labeled mice during ramp locomotion. The Ca^{2+} event activity in different trials is aligned with different movement locations or behaviors (dashed red vertical line): trial start (1st row), uphill beginning (2nd row), top of the ramp (3rd row), downhill end (4th row) and reward start (5th row). All task-related traces are averaged from all trials (24 ± 3 trials per mouse) and sorted based on their peak activation time

during the uphill beginning and displayed in temporal raster plots. Lower: averaged Ca^{2+} responses of MLIs aligned by the 5 behaviors (red curves). Gray curves indicate the Ca^{2+} responses of each animal.

(B) Activity difference between right and left trials of all MLIs in CaMKIIa–GCaMP6-labeled mice projected into 130 bins and then sorted based on their peak activation location in the L maze. In the right trials, w5 represents uphill walking and w6 represents downhill walking; opposite to the left trials. The larger proportion of MLIs showing positive activity in w5 (black rectangle) and negative activity in w6 are uphill neurons. Another group of downhill neurons is highlighted by the brown rectangle.

(C) Trial-averaged averaged MLIs activity over time of all recorded neurons sorted based on their peak activation time during four different phases (uphill, downhill, flat walking, and quiescence).

(D) Percentages of uphill, downhill, flat walking and quiescent of MLIs in the vermis of 9 CaMKIIα-transfected mice. (**p <0.01; ***p <0.001* (one-way ANOVA followed by paired Student's *t-test*, F=23.8, $p<0.001$).

Lyu et al. Figure S10

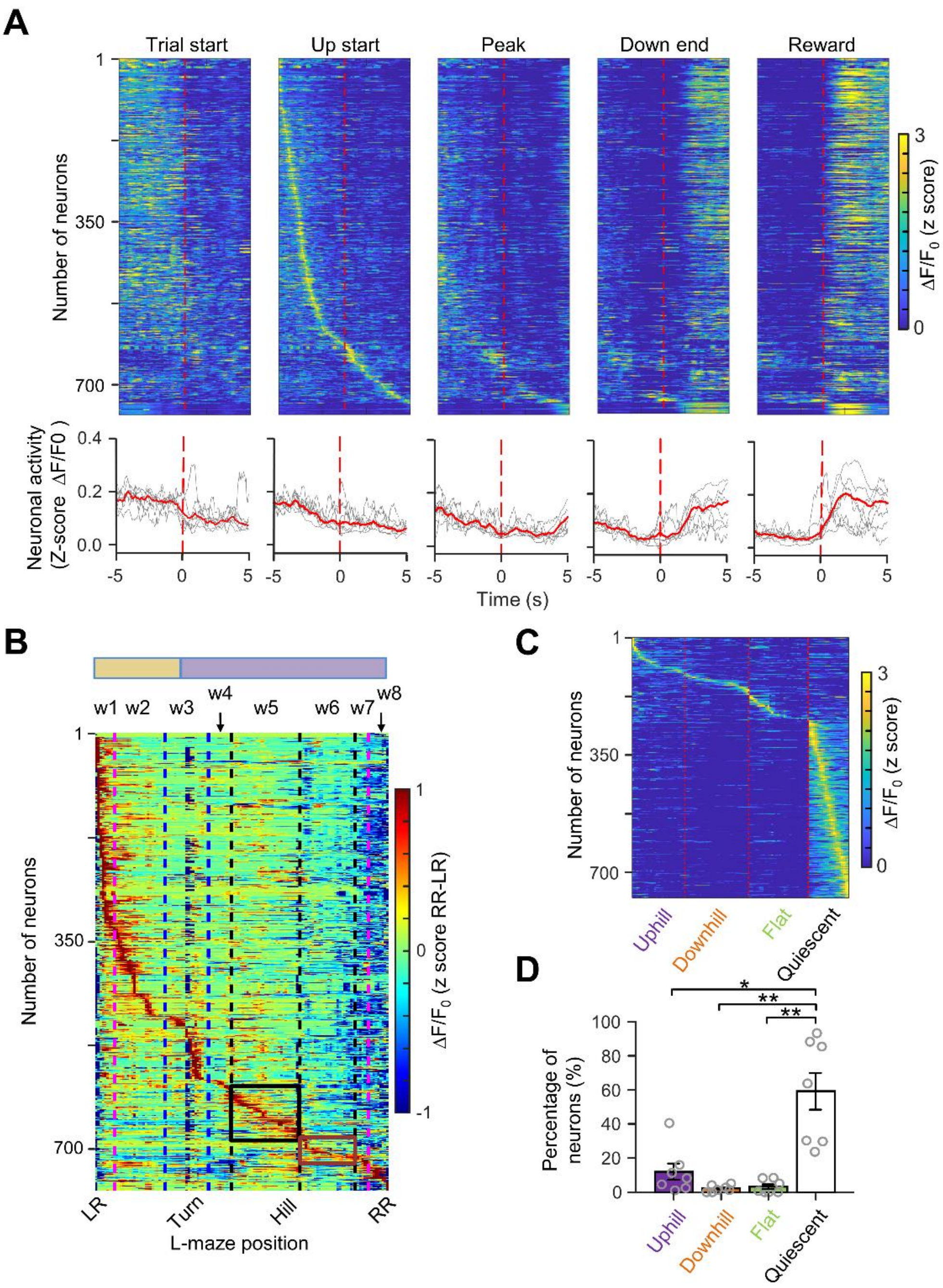

Figure S10. Vermal PCs displayed induced activity during ramp locomotion when imaging together with MLIs

(A) **Upper:** trial-averaged PCs activity of the vermis during ramp locomotion. The Ca^{2+} event activity in different trials is aligned with different movement locations or behaviors (dashed red vertical line): trial start (1st row), uphill beginning (2nd row), top of the ramp (3rd row), downhill end (4th row) and reward start (5th row). All task-related traces are averaged from all trials (24 ± 3 trials per mouse) and sorted based on their peak activation time during the uphill beginning and displayed in temporal raster plots. Lower: averaged Ca^{2+} responses of PCs aligned

by the 5 behaviors (red curves). Gray curves indicate Ca^{2+} responses of each animal.

(B) Activity difference between right and left trials of all Purkinje cells in CaMKIIα–GCaMP6-labeled mice projected into 130 bins and then sorted based on their peak activation location in the L maze. In the right trials, w5 represents uphill walking and w6 represents downhill walking; opposite to the left trials. The few Purkinje cells showing positive activity in w5 (black rectangle) and negative activity in w6 are uphill neurons. Another group of downhill neurons is highlighted by the brown rectangle. Notably, the uphill and downhill neurons are few in all recorded Purkinje cells.

(C) Trial-averaged averaged Ca^{2+} activity over time of all recorded PCs sorted based on their peak activation time during four different phases (uphill, downhill, flat walking, and quiescence).

(D) Percentages of uphill, downhill, flat walking and quiescent PCs in the vermis of 9 CaMKIIα-transfected mice. (*p <0.01; ***p <0.001 (one-way ANOVA followed by paired Student's test, $F=17.84$, $p<0.001$).

Lyu et al. Fig. S11

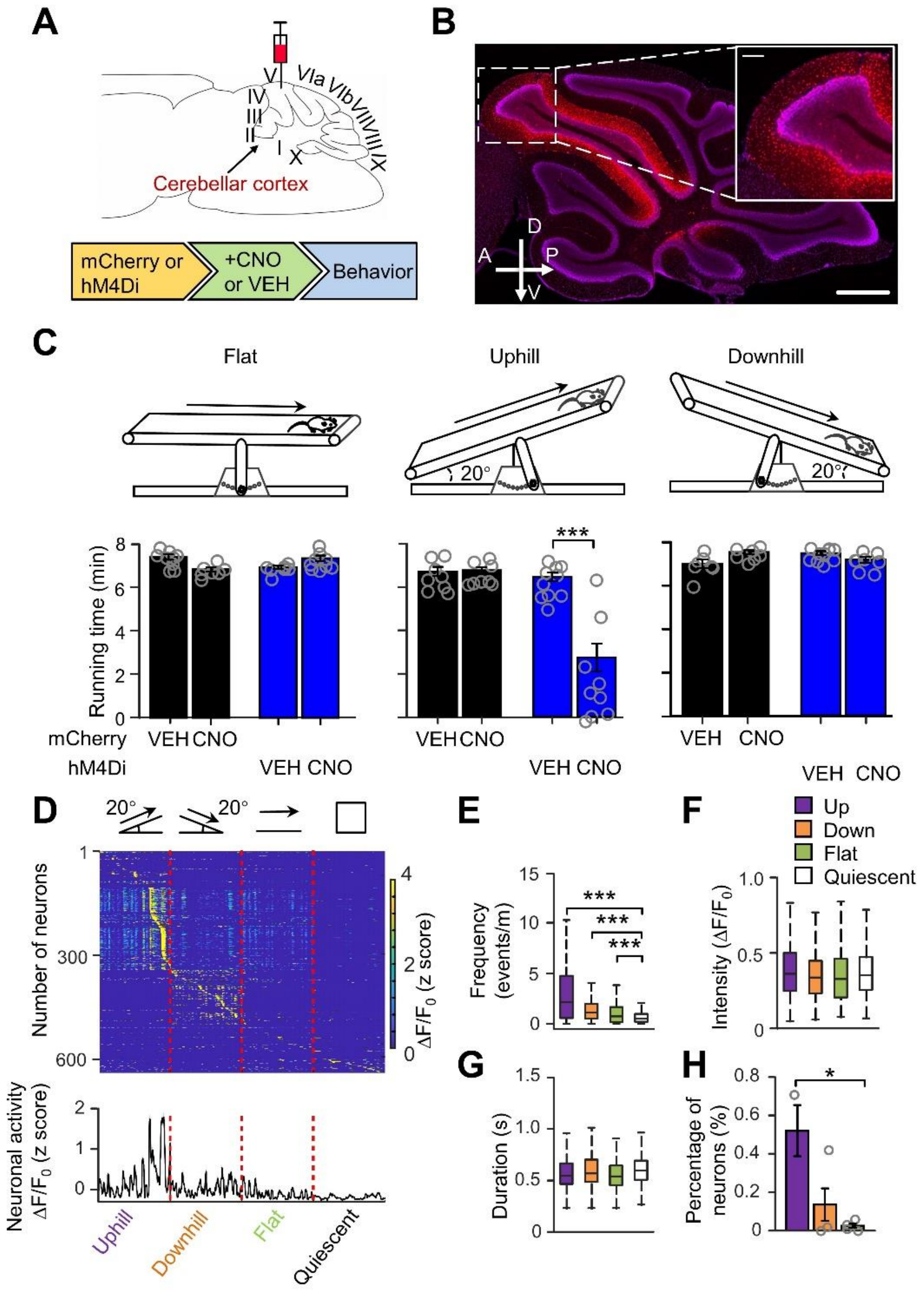

Figure S11. Cerebellar vermis is required for ramp locomotion.

(A) Upper: sketch showing the viral labeling (meCherry or hM4Di) regions in the cerebellum. Lower: outline of the experimental procedure.

(B) Confocal fluorescence image showing that lobule V is labeled by AAV-hM4Di-mecherry. Right rectangle: enlargement of dorsal lobule V. Scale bar, 500 µm.

(C) Upper: sketch showing running behavior of mice on a treadmill under flat (0º), uphill (20º), and downhill (–20º) conditions. An electric shock (1mA) was delivered to the back 1/4 of the treadmill. The task was stopped when the mice

stopped running continually for 30 seconds. Lower: bar graphs showing the running time in the flat condition (left, one-way ANOVA, F=2.49, p=0.08), uphill (middle, two-way ANOVA, F=12.15, p<0.001) and downhill (right, F=2.41, p=0.10) conditions in the same mice with hM4Di expression after CNO delivery.

(D) Ca^{2+} imaging of MLIs was performed when mice were running on the treadmill. The neuronal activity of individual MLIs was sorted based on peak activation time in four different windows (uphill, downhill, flat walking, and quiescence). Quiescence was defined as the period when the mouse rested in a small box during task change.

(E) Average Ca^{2+} event frequency (upper left, F = 218.3, $p < 0.001$) during uphill, downhill, flat walking, and quiescence in 5 mice.

(F) Average Ca^{2+} event intensity (upper right, F = 5.84, $p < 0.001$) during uphill, downhill, flat walking, and quiescence in 5 mice.

(G) Average Ca^{2+} event duration (lower left, F = 1.81, $p = 0.14$) during uphill, downhill, flat walking, and quiescence in 5 mice.

(H) Percentages of uphill, downhill, flat walking, and quiescent neurons during locomotion on the treadmill (lower right). *$p < 0.05$; **$p < 0.01$ (one-way ANOVA followed by paired Student's *t*-test, F = 6.06, $p < 0.05$).
